# Identification of long non-coding RNAs in two anthozoan species and their possible implications for coral bleaching

**DOI:** 10.1038/s41598-017-02561-y

**Published:** 2017-07-13

**Authors:** Chen Huang, Jean-Étienne R. L. Morlighem, Jing Cai, Qiwen Liao, Carlos Daniel Perez, Paula Braga Gomes, Min Guo, Gandhi Rádis-Baptista, Simon Ming-Yuen Lee

**Affiliations:** 1 State Key Laboratory of Quality Research in Chinese Medicine and Institute of Chinese Medical Sciences, University of Macau, Macau, China; 2 Laboratory of Biochemistry and Biotechnology, Institute for Marine Sciences, Federal University of Ceará, Fortaleza, Brazil; 3 Northeast Biotechnology Network (RENORBIO), Post-graduation program in Biotechnology, Federal University of Ceará, Fortaleza, Brazil; 4 Centro Acadêmico de Vitoria, Universidade Federal de Pernambuco, Vitória de Santo Antão, Brazil; 5 Departamento de Biologia, Universidade Federal Rural de Pernambuco, Recife, Brazil

## Abstract

Long non-coding RNAs (lncRNAs) have been shown to play regulatory roles in a diverse range of biological processes and are associated with the outcomes of various diseases. The majority of studies about lncRNAs focus on model organisms, with lessened investigation in non-model organisms to date. Herein, we have undertaken an investigation on lncRNA in two zoanthids (cnidarian): *Protolpalythoa varibilis* and *Palythoa caribaeorum*. A total of 11,206 and 13,240 lncRNAs were detected in *P*. *variabilis* and *P*. *caribaeorum* transcriptome, respectively. Comparison using NONCODE database indicated that the majority of these lncRNAs is taxonomically species-restricted with no identifiable orthologs. Even so, we found cases in which short regions of *P*. *caribaeorum*’s lncRNAs were similar to vertebrate species’ lncRNAs, and could be associated with lncRNA conserved regulatory functions. Consequently, some high-confidence lncRNA-mRNA interactions were predicted based on such conserved regions, therefore revealing possible involvement of lncRNAs in posttranscriptional processing and regulation in anthozoans. Moreover, investigation of differentially expressed lncRNAs, in healthy colonies and colonial individuals undergoing natural bleaching, indicated that some up-regulated lncRNAs in *P*. *caribaeorum* could posttranscriptionally regulate the mRNAs encoding proteins of Ras-mediated signal transduction pathway and components of innate immune-system, which could contribute to the molecular response of coral bleaching.

## Introduction

Advancement of high throughput sequencing technologies has allowed wide characterization of an increasing number of RNA repertoires from diverse organisms; however, only a small proportion of these effectively code for proteins and the majority remains to be studied in depth. For instance, around 1% of the human genome correlates to protein-coding transcripts, while around 4% to 9% of genome are estimated to be transcribed of which the functions are still poorly understood^1^. Due to the lack of protein-coding capacity and relatively low conservation, some of these transcripts are referred to as non-coding RNAs (ncRNAs) and at the time of their identification they were considered as “junk transcripts”^2^. Nevertheless, up-to-date in-depth analyses of ncRNAs indicated that a small proportion of these ncRNAs are implicated in a variety of biological regulations cascades and pathways, and correlated strongly with a wide range of developmental processes and diseases^3, 4^. Notably, the majority of ncRNAs discovered in recent studies had a length >200 nt, namely long noncoding RNAs (lncRNAs). Evidence has accumulated that lncRNAs could serve as key transcriptional regulators in numerous biological processes, one of the best-characterized examples of which is their role in genomic imprinting. The lncRNA namely X-inactive specific transcript (*XIST*) is responsible for inactivating the X chromosome in mammals by recruiting PRC2 (Polycomb repressive complex). In mice, *XIST* deletion causes aberrant expression of the X chromosome and female-specific lethality^5^. Another example includes p53-regulated lncRNAs in mammalian cells, i.e. *lincRNA-p21* and *PANDA*, which are capable of interacting with DNA-binding proteins and nuclear transcription factor Y alpha and suppress transcription of the target gene^6^. In addition, some investigation implicates the participation of lncRNAs in the regulation of gene expression at the posttranscriptional level. For example, an lncRNA known as cytoplasmic 1/2-sbsRNA was shown to promote mRNA decay by partial base-pairing with its specific target mRNA^7^. In another case, it was demonstrated that *lincRNA-p21* represses the translation of mRNA encoding β-catenin and JunB by partial base-pairing and recruitment of translation^8^. Apart from these examples on the function of lncRNAs at the transcriptional level of gene regulation, lncRNAs appear to serve as multi-target regulators of posttranscriptional processes, including control of mRNA splicing, degradation and translation.

Corals are among the most valuable ecosystems on Earth, providing a natural oceanic habitat for an abundance of species, ranging from microbes to vertebrates. However, over the past few decades, coral reef ecosystems worldwide are in danger due to climate changes, consequently, they are facing an unprecedented level of degradation due to the phenomena of bleaching. Bleaching is essentially defined as the loss of color, mainly caused by severe dissociation from the coral tissue of symbionts, like *Symbiodinium* ^9–11^. There are a number of factors that can trigger *Symbiodinium* escape, thus disrupting the functionality of holobionts, such as an increase in sea water temperature (global warming), marine pollution, ocean acidification and bacterial infections among others^10–12^. Several recent studies relied on massive RNA sequencing data to investigate the transcriptional changes that occur in corals in response to bleaching^13–17^. For example, Barshis and collaborators^17^ compared the gene expression among conspecific thermally sensitive and resilient corals via RNA sequencing, and found hundreds of differential expression genes (DEGs), including thermal tolerance genes and genes involved in apoptosis regulation, tumor suppression, the innate immune response, and cell adhesion. Pinzon and colleagues^13^ revealed by means of the whole transcriptome analysis that immune-related genes were differentially expressed during and after a coral bleaching event. These transcriptomic studies on the coral bleaching response focused on the changes in protein-coding RNAs, no study until now has taken into account the implication of noncoding RNAs.

In the first part of present study, we sought to identify lncRNAs in two purported congeneric species of soft coral, the zoanthids *Protopalythoa variabilis* and *Palythoa caribaeorum*. *P*. *caribaeorum* occurs in shallow waters in the western Atlantic and are relatively abundant on most coastal reefs in northeast Brazil, where they cohabits^18^. As a first step to in our survey of anthozoan lncRNAs, we employed a stringent computational filtering pipeline of transcriptomic data from deep RNA sequencing to predict with high-confidence subsets of lncRNA repertoires in these two zoanthid species. In the second part of the study, a careful examination of genes that are differentially expressed, comprising mRNAs and lncRNAs from *P*. *caribaeorum* under two physiological conditions, i.e., from healthy *P*. *caribaeorum* and from colonial individuals undergoing bleaching, was done. It is now known that episodes of bleaching occur in *P*. *caribaeorum* in the geographical localities that this species inhabit^19^. Moreover, *P*. *caribaeorum* is considered a good indicator of bleaching, since it is the first species to display the symptoms of deterioration caused by bleaching events^20^. Analytical data revealed a pattern of expression suggestive of a regulatory circuitry that operates posttranscriptionally with the participation of mRNAs and some of these novel anthozoan lncRNAs. To our knowledge, the present work is the first investigation of lncRNAs in zoanthids and their presumed regulatory role in response to coral bleaching, based on transcriptome analysis.

## Materials and Methods

### Coral sampling and sequencing

**Figure 1:**
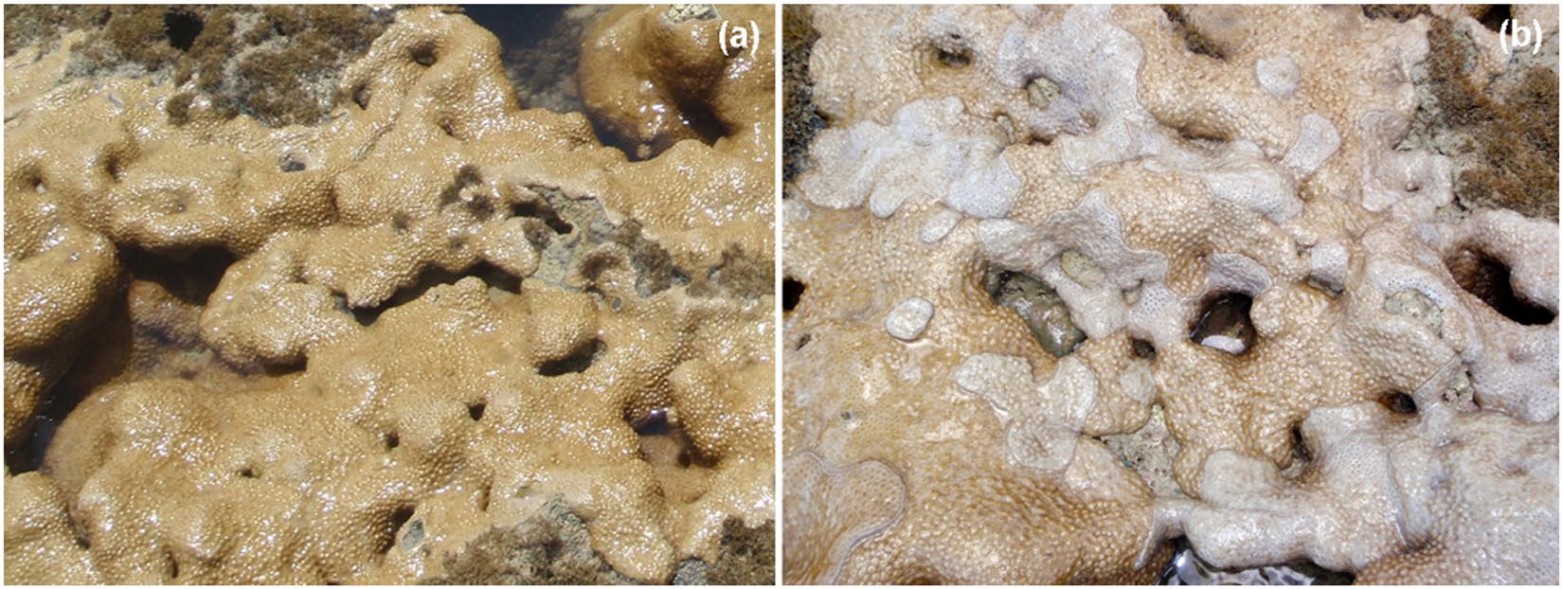
Representative images of *Palythoa caribaeorum* colonies sampled in the present study. (**a**) Healthy *Palythoa caribaeorum* colonies. (**b**) Colonies of *P*. *caribaeorum* experiencing bleaching. Both colonies were photographed on reefs of Porto de Galinhas Beach, PE, Brazil (**Photo by Dr. C. D. Perez’s research group**).

Two species of anthozoan (family Sphenopidae, order Zoantharia, subclass Hexacorallia, class Anthozoa) were investigated in the present study: *Protopalythoa variabilis* and *Palythoa caribaeorum*. Detailed sampling and sequencing information of *P*. *variabilis* were like described in our previous study (Huang *et al*.)^21^. The samples of *P*. *caribaeorum* were from healthy colonies and from colonies in the process of being bleached (Fig. 1). The tissue samples of *P*. *caribaeorum* were collected in the same geographical region and coordinate as *P*. *variabilis*, that is, they were collected in the beach-rock bands of Porto de Galinhas, Pernambuco, Brazil (8°30′20″S, 35°00′34″W). All samples were quickly chopped, transferred to 10 volumes of RNA*later* (Life Technologies, USA) and preserved at 4 °C for 48 h; subsequently, the RNA-preserving solution was then drained off for tissue storage (−80 °C) before processing. The minced tissues were powdered with a porcelain mortar and pestle under liquid nitrogen and total RNA was purified using TRIzol reagent (Life Technologies) according to the manufacturer’s protocol. The strand-specific libraries for 90 bp paired-end sequencing were prepared. Briefly, the polyadenylated RNAs (poly (A)^+^ RNAs) were isolated using oligo(dT) affinity chromatography. Single-stranded 5′-end RNA adaptors were covalently linked to mRNA fragments using T4 RNA ligase (Ambion, Austin, TX, USA) and then reversely transcribed into cDNA using Superscript III reverse transcriptase (Invitrogen, Carlsbad, CA, USA). The 3′-end DNA adaptor was ligated to the digested DNA fragments after digestion with Mmel and the products were amplified using PCR. Finally, RNA deep sequencing (RNA-seq) was conducted on a HiSeq 2500 instrument (Illumina, San Diego – CA, USA). The *P*. *variabilis* Transcriptome Shotgun Assembly (TSA) project was deposited at DDBJ/EMBL/GenBank under the accession GCVI00000000, associated with the BioProject PRJNA279783 and BioSample SAMN03450566. The TSA projects concerning to healthy *P*. *caribaeorum* and colonies undergoing bleaching were deposited under the accession GESO00000000, associated with the BioProject PRJNA320984 and BioSamples SAMN04961660 and SAMN4961665, respectively.

### Data processing, *de novo* transcriptome assembly and assessment

RNA sequencing, assembly and assessment of *P*. *variabilis* were as recently published (Huang *et al*.)^21^. In the case of the transcriptome analysis of *P*. *caribaeorum*, an individual RNA sequencing dataset (from tissue samples of healthy colonies and undergoing bleaching) and, an in-house C++ script was used to process the raw sequencing data, in order to remove low quality data (>50% of bases whose Phred scores were <5%, containing over 10% of poly-N), and the Illumina software was used to eliminate the adaptor sequences. Raw data and clean data were subjected to FastQC^22^ to evaluate sequence quality (Supplementary Figures S1–S3). Then, clean reads were used for the subsequent *de novo* transcriptome assembly process by means of Trinity^23^ with default parameters. The TIGR Gene Indices Clustering Tools (TGICL) software^24^ was used to obtain the longest and most complete consensus transcripts by clustering the assembled datasets of the two samples. Transcripts shorter than 200 nt were discarded. Furthermore, for the purpose of assessing transcriptome assembly quality as well, all reads were initially aligned to the assembled transcripts using Burrows-Wheeler Aligner (BWA) (Ver. 0.7.7-r441)^25^. Then SAMtools (Ver. 0.1.19–44428cd)^26^ and BEDTools (Ver. 2.17.0)^27^ were applied to evaluate the depth and coverage of alignment.

### Filtering pipeline for the identification of anthozoan lncRNAs

**Figure 2:**
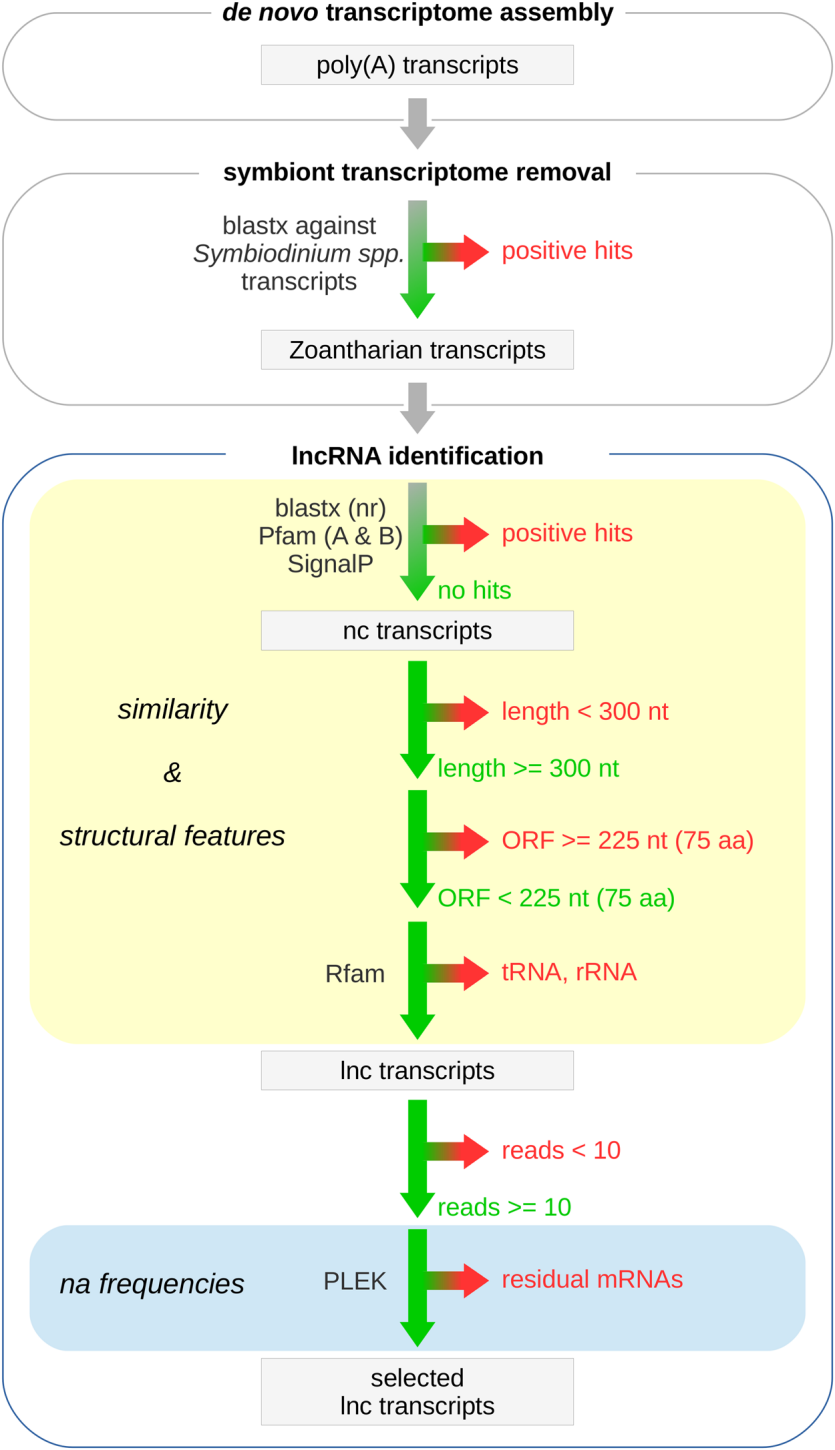
The filtering pipeline used for the identification of lncRNAs in the transcriptomes of *Protopalythoa variabilis* and *Palythoa caribaeorum*.

A stringent stepwise filtering pipeline was proposed to detect transcribed lncRNAs in both anthozoan species (Fig. 2). This pipeline is very similar to the procedures reported for the systematic screening of lncRNAs from RNA sequencing of other species, such as from the sponge *Amphimedon* ^28^ and from the plant *Panax ginseng* ^29^. Initially, all assembled transcripts were align to transcripts previously reported for the photosynthetic endosymbiont, *Symbiodinium spp*.^14^. Hits that aligned length longer than 50% of query sequences and subject sequences with an e-value less than 1E-3 were removed. This step aimed at excluding the contaminating transcriptomic sequences from endosymbiont, i.e., *Symbiodinium minutum*. It should be noted at this point that we decide not to remove endosymbiont transcriptomic sequences in the data processing step, since these sequences could be used to investigate the impact of coral bleaching on the symbiont content. Then, the remaining transcripts were subjected to the NCBI non-redundant (nr) protein database, Pfam database (both Pfam-A and Pfam-B)^30^ and Signal P4.0^31^ to search for potential protein coding transcripts. BLASTx^32^ was used to search against the non-redundant (nr) database, the E-value was set to 1e-4. For Pfam scanning and SignalP analyses, all transcripts were translated (stop-to-stop codon) using in-house perl script, and the longest ORF for each transcript was retained. Transcripts returning at least one hit by one of the three search methods were removed. To reduce the number of potential spurious transcripts found in transcriptome assemblies, transcripts shorter than 300nt were also removed^28^. In addition, only the remaining transcripts for which the largest predicted ORF corresponded to precursors of no more than 75 amino acid residues (aa) were considered as lncRNA candidates. Next, transcripts annotated as housekeeping non-protein coding RNAs (npcRNAs), such as tRNAs and rRNA were excluded from the remaining lncRNA candidates by subjecting them to the Rfam database^33^. Additionally, only lncRNA candidates with an overall expression of at least 10 raw read counts were retained. Finally, a tool for predicting lncRNAs and mRNAs, PLEK^34^ was used to evaluate the sensitivity of the bioinformatic pipeline. Only transcripts that were classified as noncoding by PLEK (Ver. 1.2 were identified as high-confidence lncRNAs.

### Differential expression analysis of anthozoan lncRNAs and mRNAs

Clean reads from one-stranded paired-end libraries of *P*. *variabilis* and two-stranded paired-end libraries of *P*. *caribaeorum* were mapped to the corresponding assembled transcripts using the Burrows-Wheeler transform, BWA (-o 1 -e 50 -i 15 -L -l 31 -k 2 -t 4)^35^. The number of reads that mapped to each transcript were calculated using in-house Perl script, and fragments per kilobase of transcript per million mapped reads (FPKM)^36^ of transcripts of each sample were also calculated by in-house Perl script. The transcripts were identified as differential expression genes when the absolute value of Log_2_ (FPKM1/FPKM2) ≧1, at a 5% false discovery rate (P value adjusted for multiple testing using the Benjamini–Hochberg correction).

### KEGG pathway analysi

Ingenuity pathway analysis was used to identify enriched metabolic and/or signal transduction pathways related to significantly differentially expressed mRNAs of *P*. *caribaeorum*, according to the Kyoto Encyclopedia of Genes and Genomes (KEGG). This approach mapped firstly all DGEs concerned with the proteins of KEGG, and then found significantly enriched pathways. A strict algorithm was proposed, described as follows,

$$P=1-\sum _{i=0}^{m-1}\frac{(\begin{array}{c}M\\ i\end{array})(\begin{array}{c}N-M\\ n-i\end{array})}{(\begin{array}{c}N\\ n\end{array})}$$

where *N* is the total number of genes with KEGG annotation, n is the number of DEGs in *N*, *M* is the total number of genes annotated to specific pathways, and m is the number of DEGs in *M*. The calculated p-value was subjected to the Bonferroni Correction, with the taking corrected p-value ≧ 0.05 taken as the threshold for significane.

### GO annotation analysis

All of the differential expression mRNAs were first searched using BLASTx against the NCBI non-redundant (nr) protein database with a cut-off E-value of 10^−3^. Then, all the blast results were subjected to the GO database to retrieve GO annotation information using Blast2GO^37^. Finally, the annotation results were visualized using the WEGO (Web Gene Ontology Annotation Plot) tool^38^.

### Target mRNAs for prediction of *P. caribaeorum* lncRNAs

In this study, which aimed to identify target mRNAs for novel anthozoan lncRNAs, as well as to correlate lncRNA functions to physiopathological responses to coral bleaching at the molecular level, we focused our analysis on the lncRNA repertoire of *P*. *caribaeorum*. Firstly, all *P*. *caribaeorum* lncRNAs were compared with known lncRNAs of the NONCODE database using BLASTn, the E-value was set to 1e-3 (for differentially expressed lncRNAs, E-value = 1.0). Aligned regions between queried lncRNAs and target lncRNA sequences in NONCODE were regarded as putative functional conservative regions. Secondly, the aligned lncRNAs were subjected to RNAplex to search for probable lncRNA-mRNA interactions among the 54,699 known mRNAs of *P*. *caribaeorum* transcriptome, using default parameters, but with the exception that the temperature of simulative hybridization was set to 25 °C. RNAplex is a tool especially designed to search for short segments of interactions between two long RNAs^39^. It can rapidly compute optimal secondary structures for their hybridization based on free energy minimization. The mRNA-lncRNA interactions in which hybridization sites were located in conservative regions were selected for further investigation. Finally, the mRNAs that were shown to interact with *P*. *caribaeorum* lncRNAs in a relatively perfect complementary base-pairing manner on the conservative regions, and having lower interaction energy (≤−20) requirements, were considered as being high-confidence target mRNAs for their corresponding lncRNAs. More specific details of lncRNAs and mRNAs interactions in *P*. *caribaeorum* are presented in the results.

### Statistical analysis

The Kolmogorov-Smirnov test (KS test), a nonparametric test of the equality of continuous, one-dimensional probability distributions, is one of the most useful and widely used nonparametric methods for comparing two samples, as it is sensitive to differences in both the location and shape of the empirical cumulative distribution functions of two samples. In the present study, we used the two-sample KS test to evaluate the significance of differences between mRNAs and lncRNAs among two coral species. The whole statistical analyses are performed using the R package.

## Results

### Data processing, transcriptome assembly and identification of lncRNAs in *P. variabilis* and *P. caribaeorum* transcriptomes

RNA sequencing data processing, and assembly and assessment of *P*. *variabilis* transcriptome were conducted as detailed in our previous study^21^. Briefly, a total of 60,891,368 clean reads were assembled into 126,441 transcripts. Sequence alignment of clean reads against assembled transcripts using BWA indicated that 58,916,865 (87.22%) reads could be mapped to the assembled transcripts. Furthermore, assessment of the depth and coverage of alignment, done using SAMtools and BEDTools showed that 121,877 transcripts were mapped to at least 10 reads, in which more than 80% of the sequences were covered by reads.

In the case of *P*. *caribaeorum* transcriptomes, deep RNA sequencing of paired-end 90 nt was conducted with samples from the tissues of healthy colonies and from individuals undergoing bleaching, resulted in a total of 63,914,343 and 55,523,043 reads, respectively. The sequence quality analysis of clean data for both samples is presented in Figures S2 and S3. Following this assessment, the transcripts of each sample were firstly assembled using Trinity and then clustered together using TGICL to obtain the consensus transcript sequences. At the end of the process, a dataset of 136,654 transcripts with a mean sequence length of 874 nt, was obtained for the combined transcriptomes of healthy *P*. *caribaeorum* tissue and tissue undergoing bleaching. Sequence alignment of clean reads of healthy *P*. *caribaeorum* against assembled transcripts using BWA indicated that 57,291,678 (89.63%) reads could be mapped back to the assembled transcripts. Furthermore, the depth and coverage assessment of alignment using SAMtools and BEDTools showed that 123,675 transcripts were mapped to at least 10 reads, in which more than 80% of sequences were covered by reads. On the other hand, 48,678,012 (86.90%) of the reads of *P*. *caribaeorum* experiencing bleaching could be aligned to the assembled transcripts and 118,345 transcripts were mapped at least ten reads and in which more than 80% of sequences were covered by reads.

In order to identify the putative lncRNAs transcribed in *P*. *variabilis* and *P*. *caribaeorum*, all assembled transcripts were subjected to a stringent stepwise filtering pipeline (Fig. 2). Three core filtering criteria were applied to screen these anthozoan lncRNAs: (1) the potential of encoding proteins; (2) the length of transcripts; and (3) the size of the open reading frames (ORFs). Taking the dataset from *P*. *variabilis* as an example of our detailed filtering process, a total of 20,400 transcripts that aligned to *Symbiodinium* transcript sequences were initially removed. Thereafter, the remaining zoanthid (*P*. *variabilis*) transcripts were searched for sequence similarity with known proteins in the NCBI non-redundant database, for finding functional domains in the Pfam database and predicted leader sequences (signal peptides), which were also removed. Subsequently, transcripts shorter than 300 nucleotides, a stricter cutoff than the 200 nt commonly used, were filtered out. By means of these steps, 32,028 (ncRNAs) were retained. Next, the ncRNAs were subjected to the ORF prediction and subsequently filtered by removing the transcripts of protein-coding potential, based on a maximum ORF size of 75 amino acids.

The remaining ncRNAs were again filtered to eliminate housekeeping ncRNAs, like tRNAs and rRNAs, based on Rfam scanning. This approach led to the retention of 11,453 lncRNA candidates. Subsequently, to reduce noise without losing low-abundance transcripts, the lncRNA candidates with an overall expression of less than 10 raw read counts were removed as in a previous study by Gaiti *et al*.^28^. The cross-validation of these lncRNA candidates, using PLEK software and a comprehensive stringent pipeline, identified a final set of 11,206 lncRNAs in the *P*. *variabilis* transcriptomic dataset. A similar number of lncRNA candidates, i.e., 13,240 lncRNAs, were also identified in healthy *P*. *caribaeorum* transcriptomes.

### Features and functional analysis of lncRNAs from *P. variabilis* and *P. caribaeorum* transcriptomes

**Figure 3:**
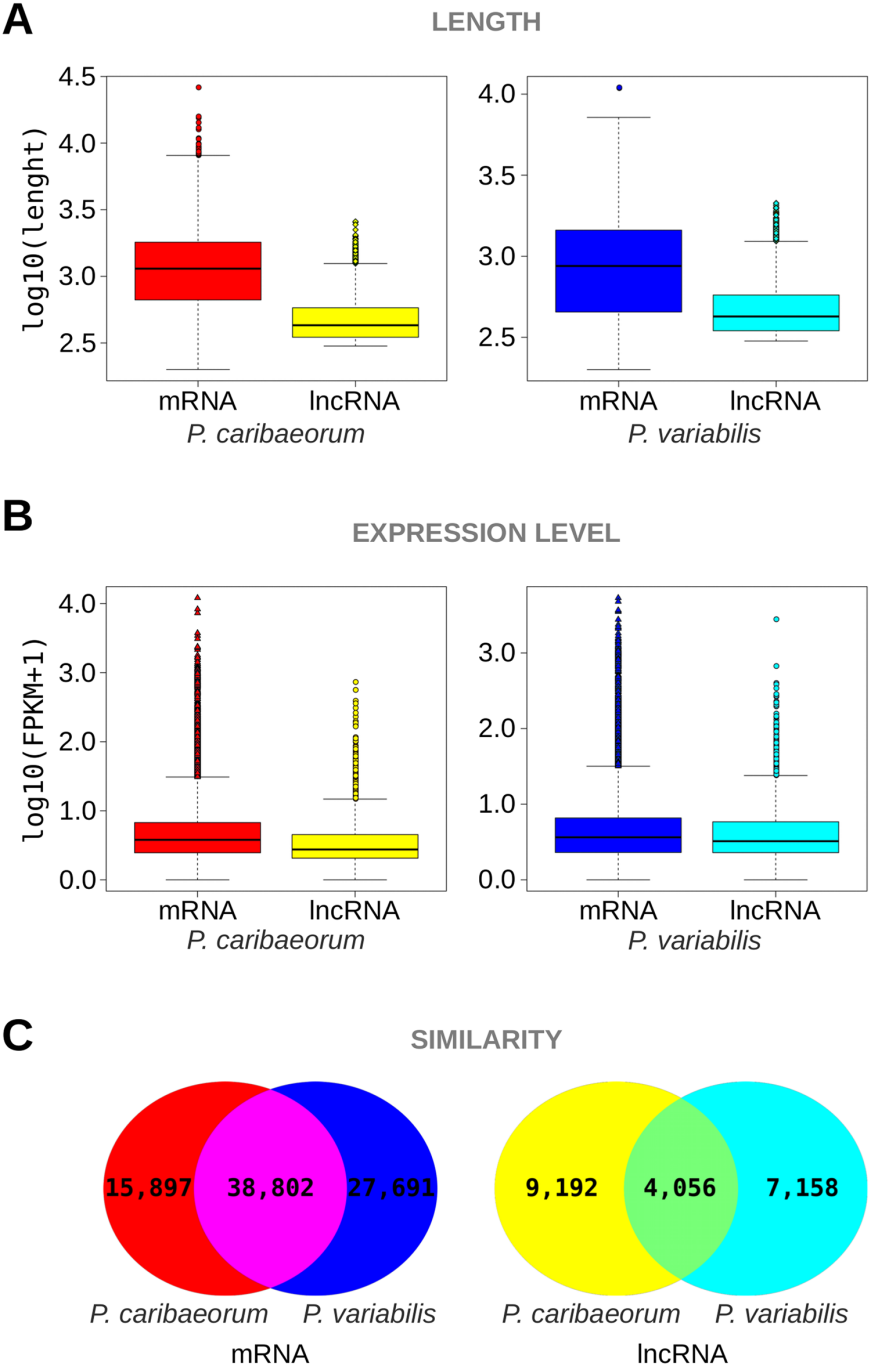
General characteristics of mRNAs and lncRNAs in *Protopalythoa variabilis* and *Palythoa caribaeorum*. (**A**) Distribution of transcript length by log_10_ (Length) in mRNAs and lncRNAs in *Protopalythoa variabilis* and *Palythoa caribaeorum*. (**B**) Expression level indicated by log_10_ (FPKM + 1) in mRNAs and lncRNAs in *Protopalythoa variabilis* and *Palythoa caribaeorum*. (**C**) Comparison of mRNAs and lncRNAs from *P*.*variabilis* and *P*. *caribaeorum*.

As mentioned above, a total of 11,206 and 13,240 lncRNAs were identified in the transcriptome of *P*. *variabilis* and healthy *P*. *caribaeorum*, respectively. In order to comprehensively examine the differences and similarities between mRNAs and lncRNAs, comparative analysis was performed based on the lengths and structures of transcripts, as well as their expression levels. The results showed that the length of mRNAs of both species of anthozoan was greater than that of their respective lncRNAs (Kolmogorov-Smirnov test, P < 0.0005) (Fig. 3A). Moreover, lncRNAs in both anthozoans showed a lower level of expression when compared to the respective levels of mRNA expression (Kolmogorov-Smirnov test, P < 0.0005) (Fig. 3B). Additionally, we assessed the similarity of the mRNAs and lncRNAs repertoires of *P*. *variabilis* and healthy *P*. *caribaeorum* using BLASTn. For this purpose, a relatively strict criterion to indexing similarity was defined: the matching regions should be longer than half the size of any compared lncRNAs. The results indicated that 3,469 lncRNAs (about a third of the predicted lncRNAs) shared a high similarity (79~100% identities) between lncRNAs from *P*. *variabilis* and healthy *P*. *caribaeorum* (Fig. 3C). By contrast, 38,802 mRNAs (more than 50% of the predicted coding transcripts) of *P*. *variabilis* were shown to be homologous to their transcript counterparts in *P*. *caribaeorum* (Fig. 3C). The sequence conservation observed in the collection of lncRNAs and mRNAs from both anthozoans is justifiable and corroborated by the fact that both species are suggested to be congeneric^40^. Importantly, some lncRNAs from these two anthozoan repertoires, as the long intervening non-coding RNAs (lincRNAs), were found to have sequences less conserved than mRNA sequences, consequently being more lineage-specific as observed for other species^41^.

#### Ultra-conserved regions exist in lncRNAs from *P*. *variabilis*, *P*. *caribaeorum* and model species in the NONCODE database

To address the functionality of lncRNAs, all putative lncRNAs of *P*. *variabilis* and *P*. *caribaeorum* were compared with known lncRNA sequences in the NONCODE v3.0 database using BLASTn, with the E-value adjusted to 1e-3. The result showed that approximately 1% of *P*. *variabilis* and *P*. *caribaeorum* lncRNAs are structurally comparable with lncRNA sequences from diverse vertebrate species (Figure S4). This low percentage was actually expected, since a low number of lncRNA sequences are shared among species, as observed in zebrafish and human (6%)^42^. Noteworthy, among the compared lncRNA sequences and in contrast to protein-coding RNAs, in which long segments are typically very similar to their homologues, most of lncRNAs shared a common sequence only for short stretches of 10 bp to 50 bp. For instance, comparison of a lncRNA of *P*. *caribaeorum*, namely CL4490. Contig 2, to lncRNA sequences from human, chimpanzee and mouse, showed that lncRNA CL4490. Contig 2 displays relatively low similarity with the corresponding short (divergent) regions of lncRNAs from these higher vertebrate species (Figure S5). A possible explanation for this structural characteristic of lncRNAs is that they are quite distinct from mRNAs in that mRNAs have to conserve codon integrity and prevent frameshift mutations in a single long ORF. In addition, lncRNAs are subjected to a relatively low number of evolutionary constraints, with the exception of a selective pressure to strictly conserve the short lncRNA regions, responsible either for sequence-specific interactions or structural organization. Indeed, in previous studies on lncRNAs, it was demonstrated that a small proportion of lncRNAs from mammals and zebrafish retains interspecies short and highly conserved regions^42, 43^. Moreover, antisense reagents targeting these conserved regions in zebrafish lncRNAs resulted in developmental defects^42^, suggesting that the conserved regions play a crucial role in lncRNAs functions, i.e., in the regulation of gene expression, via hybridizing with transcriptional elements, such as promoter, transcription factor, repressor, and/or enhancer.

#### Prediction of *P*. *caribaeorum* lncRNAs function based on ultra-conserved regions

**Figure 4:**
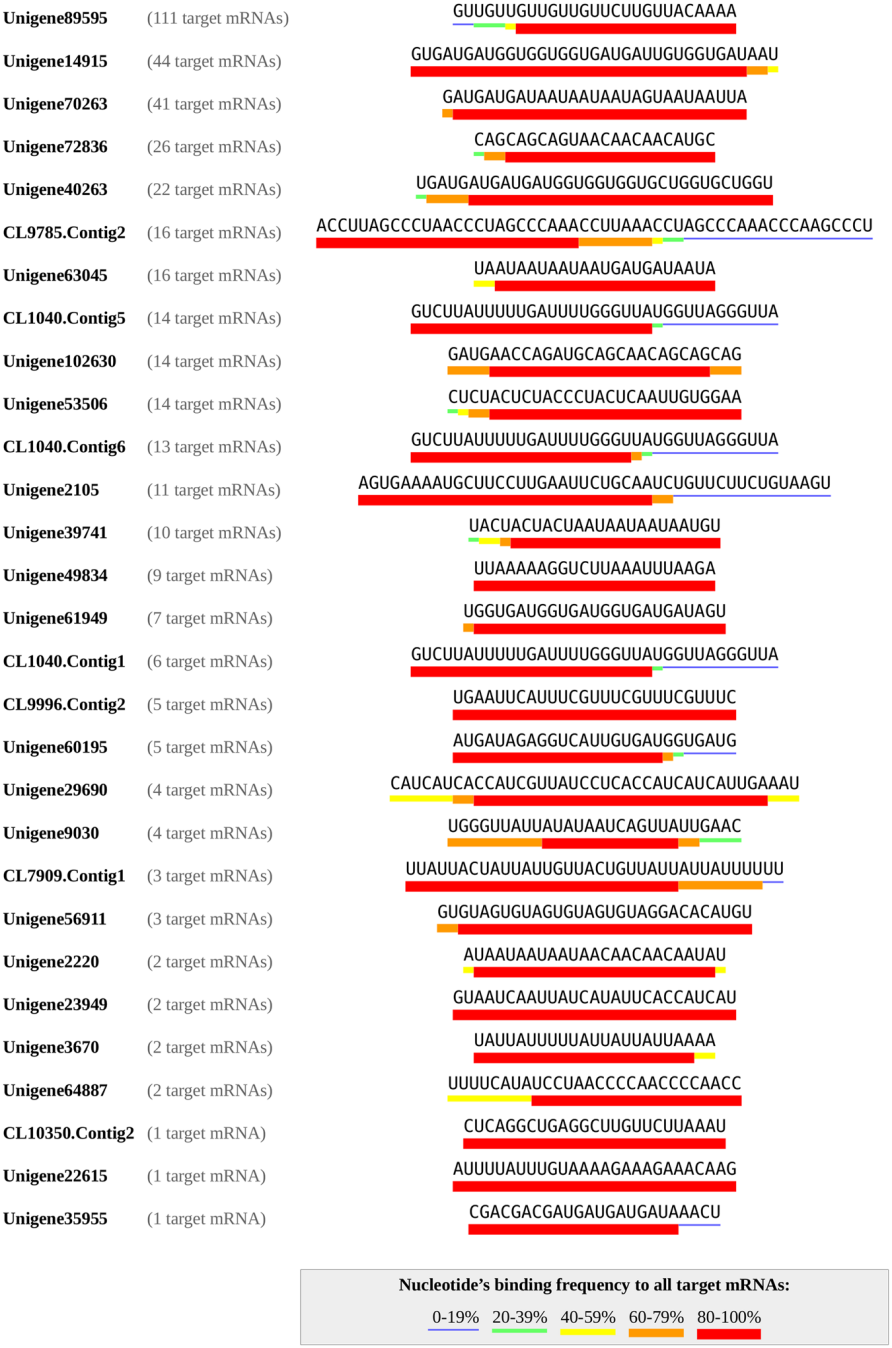
Inter-species conserved region of 29 lncRNAs from *Palythoa caribaeorum*, involved in the predicted lncRNA-mRNA interactions. Different color bars below the conserved region sequence show each nucleotide’s frequency of binding to all target mRNAs, blue: 0–19%, green: 20–39%, yellow: 40–59%, orange: 60–79%, red: 80–100%.

It is rationally and intuitive to suggest that conserved regions in lncRNAs would be retained over the long course of the evolutionary history of distant parental organisms only if those conserved sequences, if altered, could critically affect key components of cellular processes. Several current studies about lncRNAs have elucidated the diverse range of functions mediated by lncRNA, based mainly on the intermolecular RNA-RNA and RNA-DNA interactions, complementary base-pairing, or the formation of an RNA-protein complex via recruitment of specific proteins^6^. Hence, we reasoned that such conserved regions in anthozoan lncRNAs could interact with RNA, DNA or protein target and also exert their regulatory effects also in zoanthids. To assess this, a total of 145 lncRNAs from healthy *P*. *caribaeorum*, with conserved regions highly comparable to those of lncRNAs found in other organisms were considered here for further investigation. Those lncRNAs were subjected to RNAplex to search for probable lncRNA-mRNA interaction with 54,699 identified *P*. *caribaeorum* mRNAs. RNAplex tools are allowed identification of the optimal hybridization sites for each queried lncRNA-mRNA pair, taking into account the minimization of free energy. The aim of this step was to examine if conserved regions tend to be located in the hybridization sites of lncRNA-mRNA interaction. The results of the predictive analysis showed that a large proportion of lncRNAs (around 90%) could hybridize and interact with target mRNAs in their respective matched conserved regions (Supplementary Table 1), lending support to a possible regulatory role of these lncRNAs in *P*. *caribaeorum* tissue. Of all of these possible interactions, 29 lncRNAs, in particular, have been shown to interact with the conserved region of 371 target mRNAs in a complementary base-pairing manner and with a low predicted energy (≤−20) (Fig. 4, example in Figure S6), which forms a 402 mRNAs-lncRNAs interaction network. The majority of the mRNA-lncRNA interactions were, however, predicted to hybridize defectively in terms of base complementarity (Supplementary Table 1). In fact, it is not surprising to see such defective base pairing, since most of the ncRNAs essentially tend to interact in an intricate way which does not necessarily involves accurate hybridization^44^. Furthermore, around 10% lncRNAs were found to not predictively any kind of interaction with the same set of mRNAs (Supplementary Table 1), probably because conserved regions of these lncRNAs might specifically bind to special elements other than target mRNAs, such as certain DNAs or proteins. Obviously, for definitive conclusions about lncRNA target-interactions and functionality to be drawn, further ingenious experimental design and validation is required.

#### Functional classification of target mRNAs potentially regulated by *P*. *caribaeorum* lncRNAs

**Figure 5:**
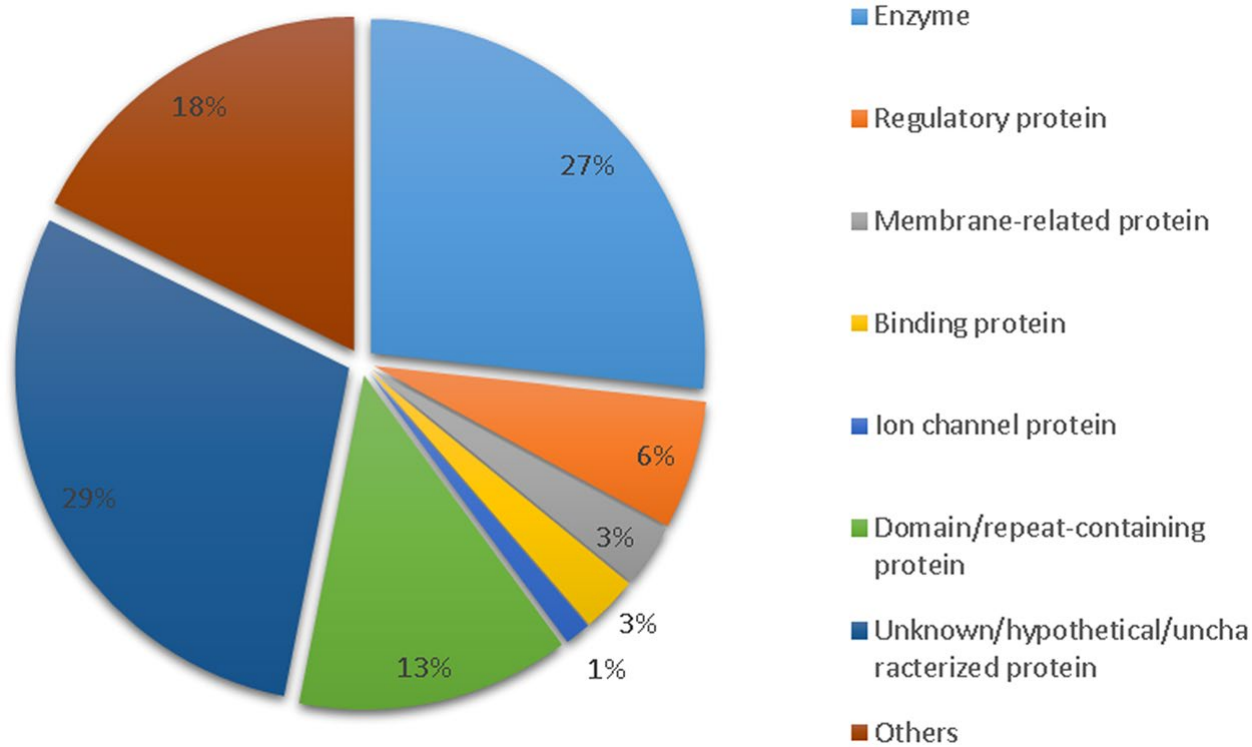
Functional classification of interacting mRNAs targeted by 29 lncRNAs from *P*. *caribaeorum*. Functions of target mRNAs were extracted based on the annotation information of BLAST alignment against the NR database. The lncRNA-mRNA interactions not only contain relatively perfect complementary base-pairing, but also require low interaction energy (≤−20) for hybridization.

**Figure 6:**
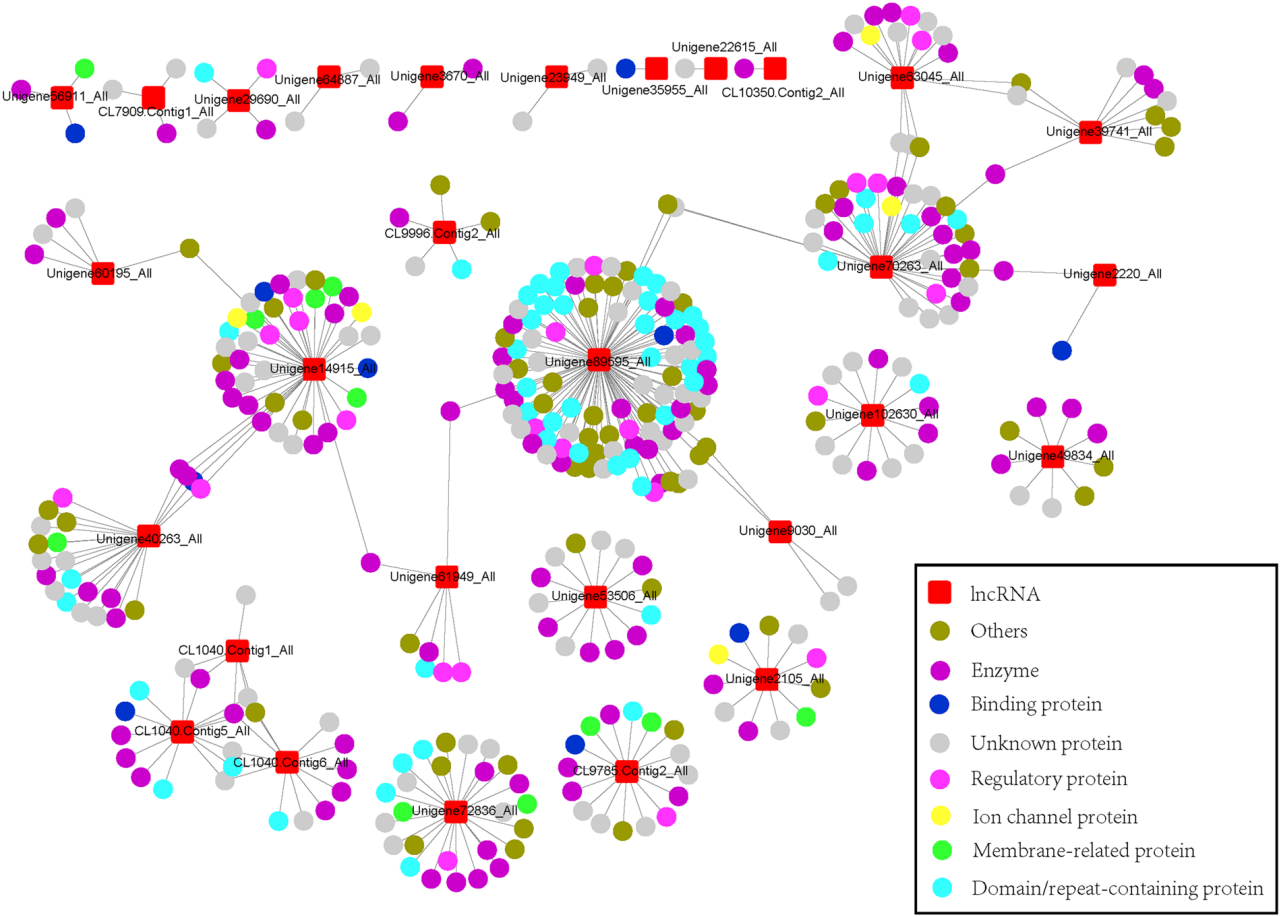
lncRNA-mRNA interaction network constructed based on the 29 lncRNA-mRNA interaction pairs. Target mRNAs were classified based on coding proteins annotated from BLAST alignment against the NR database. The lncRNA-mRNA interactions not only contain relatively perfect complementary base-pairing, but also require low interaction energy (≤−20) for hybridization.

Based on the annotation by BLASTx against the nr database, target mRNAs that could be posttranscriptionally regulated by the novel 29 lncRNAs of *P*. *caribaeorum* were classified into eight categories (Fig. 5). Specifically, these 29 lncRNAs were predicted to interact with, and potentially regulate mRNAs encoding proteins that are involved in multiple processing networks of biological activity and cellular metabolism (Fig. 6 and Table S2). For instance, some of these interactions were observed to predictively target mRNAs encoding various enzymes involved in RNA transcription, and DNA assembly, replication and modification, including enzymatic components such as transcriptase, exoribonuclease, DNA polymerase, and nuclease, etc. In addition, around 6% of lncRNAs were predicted to interact with mRNAs encoding regulatory proteins (Fig. 5), such as transcription elongation regulator, translation initiation factor, nuclear factor, transcription factor, splicing factor, nonsense transcript regulator and transcription complex subunits. Concerning this aspect of DNA and RNA metabolism, it is now known and accepted that lncRNAs can work as transcriptional regulators by interacting directly with transcription factors, acting on promoters and repressors for activation or inactivation of gene expression^45^. Our findings reveal that lncRNAs in *P*. *caribaeorum* may be involved in regulation at the posttranscriptional level of key proteins involved in RNA metabolism in *P*. *caribaeorum* cells. Another small set of 29 *P*. *caribaeorum* lncRNAs (Table S2) was predicted and found to only target mRNA sequences encoding several unknown or hypothetical proteins, although they were specifically encoded by a marine cnidarian model, the starlet sea anemone *Nematostella vectensis*. It is worthy of noting that the predicted interaction of *P*. *caribaeorum* lncRNAs with mRNAs encoding unknown/hypothetical protein sequences in *N*. *vectensis*, suggests a cross-species regulation of a common group of target transcripts in these distant but closely related species of cnidarians.

### Differential expression of mRNAs and lncRNAs in *P. caribaeorum*: a clue to the possible implications of coral bleaching?

#### Identification of differentially expressed mRNAs and lncRNAs in healthy *P*. *caribaeorum* tissues and those undergoing bleaching

**Figure 7:**
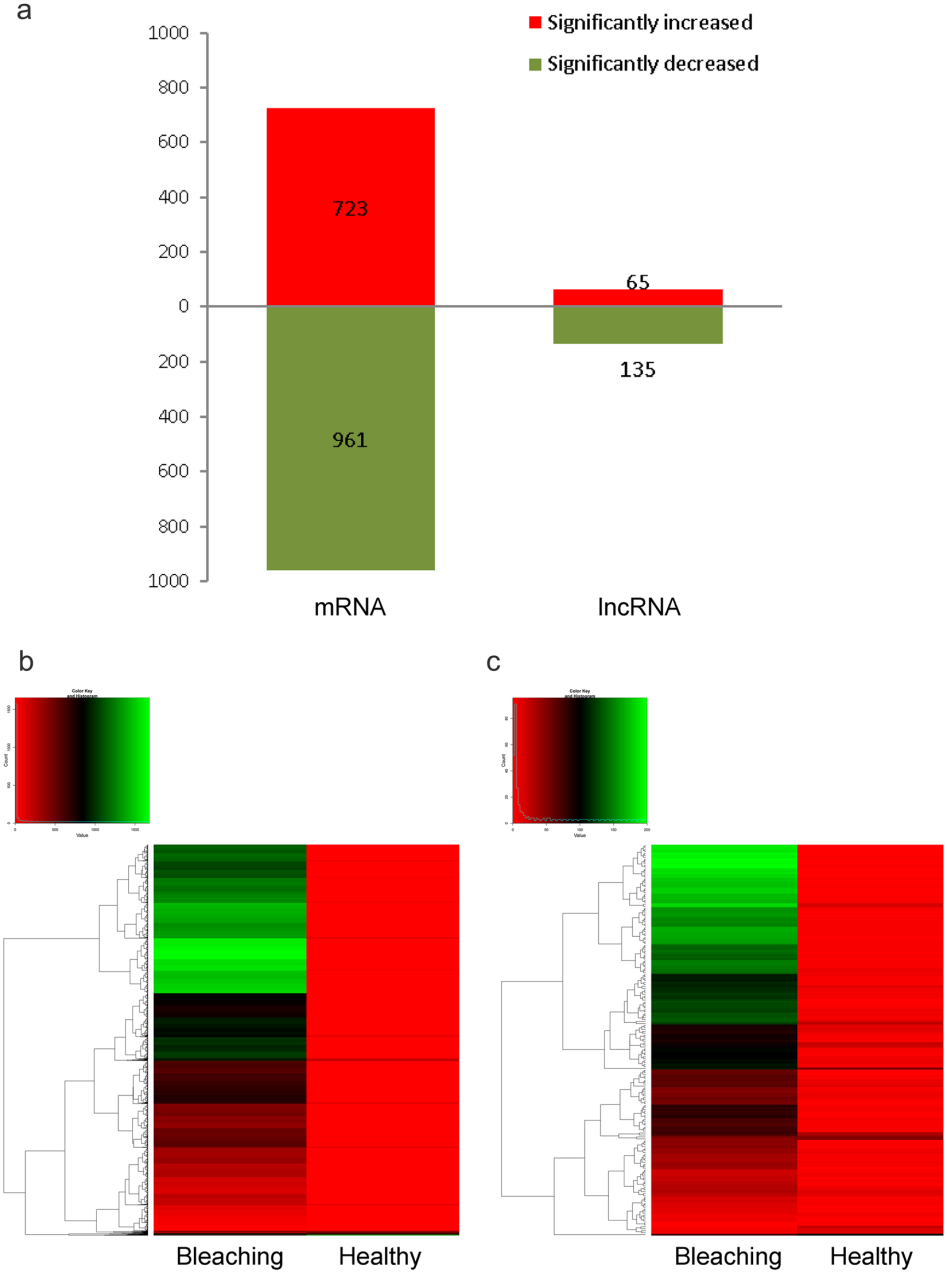
Comparison of differentially expressed mRNAs and lncRNAs identified in healthy *P*. *caribaeorum* and in colonial individuals undergoing bleaching. (**a**) Histogram plot of significantly differentially regulated mRNAs and lncRNAs in *P*. *caribaeorum* tissue undergoing bleaching compared to healthy tissue. (**b**,**c**) Hierarchical clustering and scatter plot of overall mRNAs (**b**) and lncRNAs (**c**) differentially expressed in *P*. *caribaeorum* tissue undergoing bleaching compared to healthy tissue. Red: lower expression levels, green: higher expression levels.

Comparison of the transcriptomes of *P*. *caribaeorum* in two different physiological states, i.e., from tissues of a healthy colony and from a colony going through the process of bleaching, has allowed us to disclose transcribed genes and pathways seemingly associated with the coral bleaching response. A total of 1,684 mRNAs and 200 lncRNAs were identified in *P*. *caribaeorum* by DGEs analysis after transcriptome assembly and sequence alignments (Fig. 7). Of 1,684 identified mRNAs, 723 were up-regulated and 961 were down-regulated (fold-change >2, P < 0.05) in *P*. *caribaeorum* undergoing bleaching compared to healthy *P*. *caribaeorum*, respectively (Fig. 7a). Of 200 differently expressed lncRNAs, 65 were up-regulated and 135 were down-regulated (fold-change >2, P < 0.05), in *P*. *caribaeorum* undergoing bleaching versus healthy *P*. *caribaeorum*, respectively (Fig. 7a). The distinguishable mRNA and lncRNA expression profiles of healthy *P*. *caribaeorum* and those undergoing bleaching are depicted in Figure 7b,c.

#### KEGG pathway enrichment analysis of differentially expressed mRNAs

**Figure 8:**
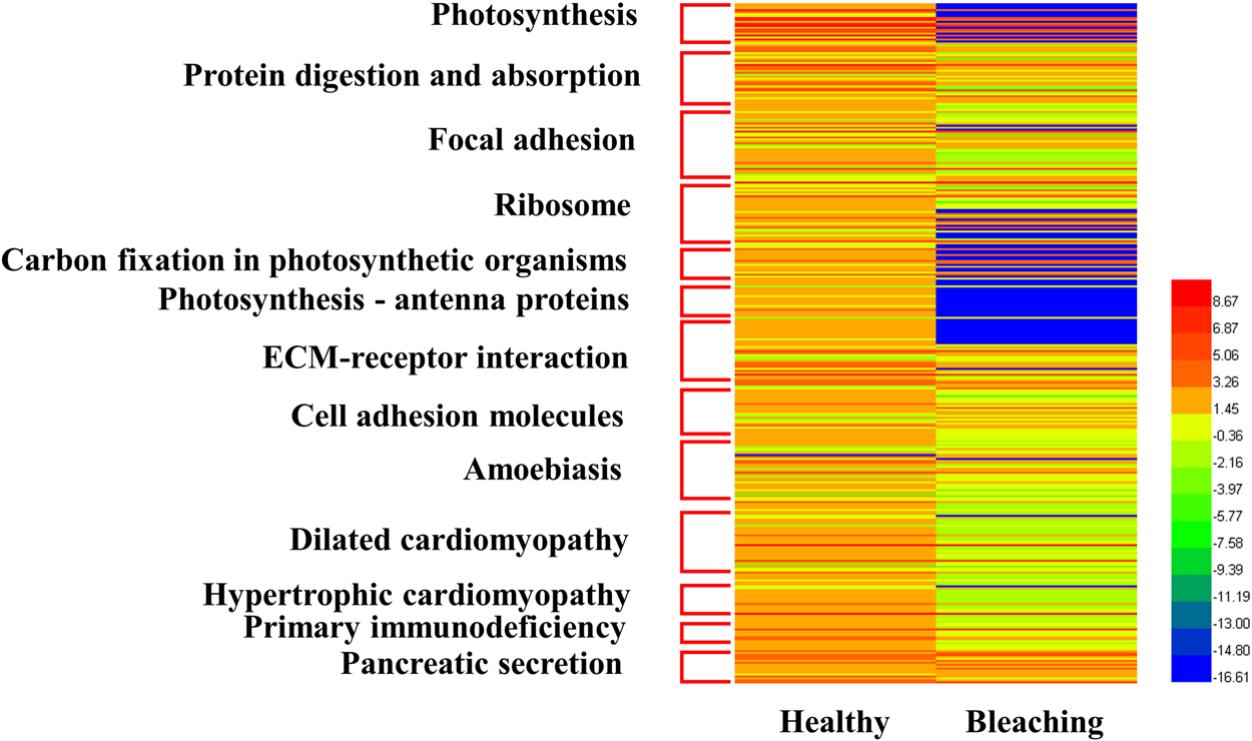
Heatmap of 347 differentially expressed mRNAs enriched to 13 KEGG pathways in *P*. *caribaeorum* tissue undergoing bleaching compared to healthy tissue. Expression levels were normalized by logarithmic base 2.

**Figure 9:**
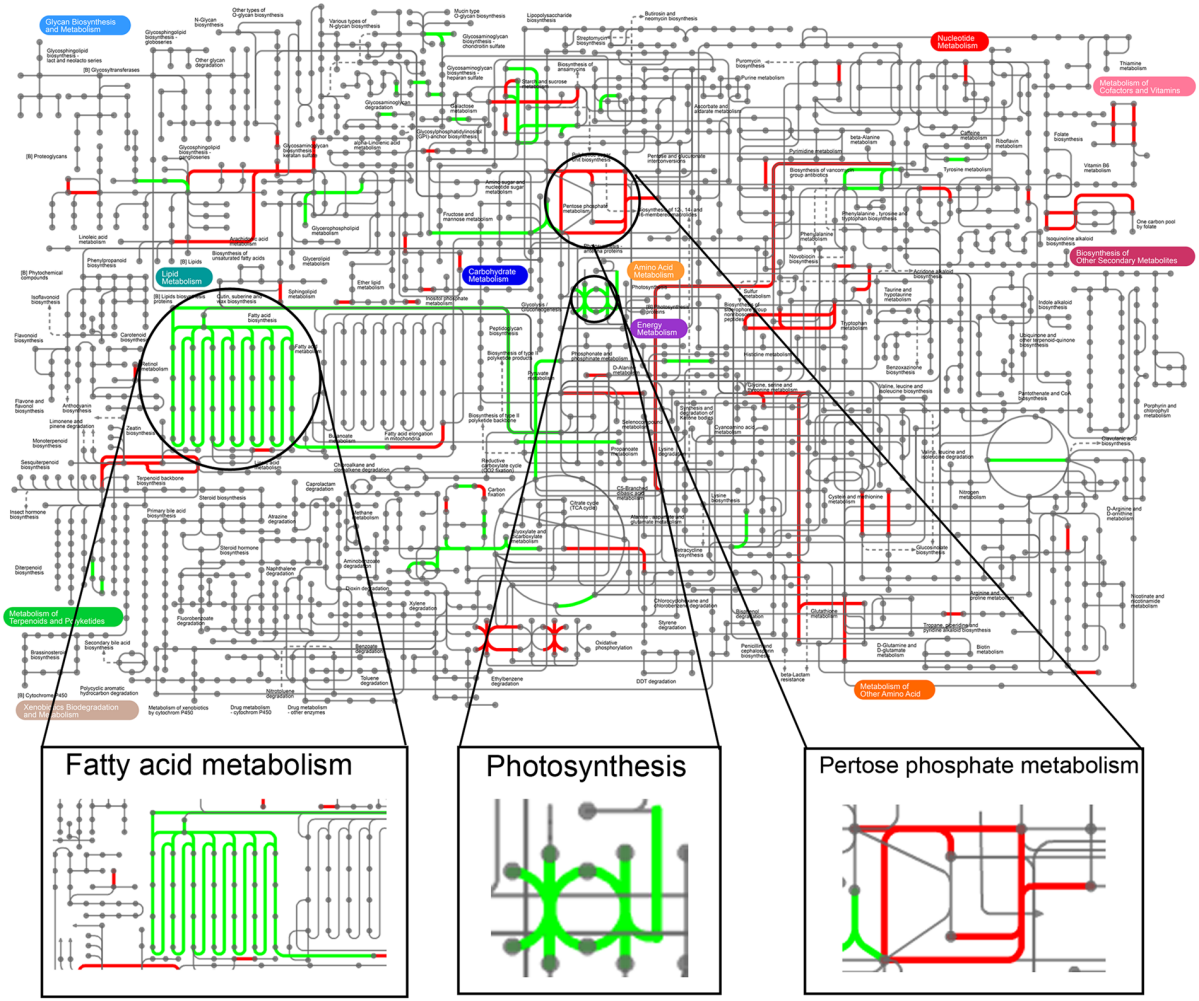
Visualization of differentially expressed mRNAs enriched in the metabolic pathways of *P*. *caribaeorum*. All differentially expressed mRNAs were subjected to the web-based tool, IPath2.0^69^ for visualization. Up-regulated genes are highlighted in red, down-regulated genes are highlighted in green.

KEGG enrichment analysis indicated that the major pathways via which transcripts were differentially expressed in *P*. *caribaeorum* going through the process of bleaching are associated with energy metabolism, cell adhesion and immunity, including photosynthesis, protein digestion and absorption, focal adhesion, ribosome biogenesis, carbon fixation in photosynthetic organisms and photosynthesis-antenna proteins, among others (Table 1). Indeed, the overall expression level of differentially expressed mRNAs mapped to KEGG pathways was found to decrease by different amounts in *P*. *caribaeorum* in undergoing bleaching (Fig. 8), particularly in respect to pathways related to energy metabolism, like photosynthesis and energy capture by antenna proteins, and photosynthetic carbon fixation (Table 1 and Fig. 9). These findings could be reasonably explained by the fact that coral host undergoing bleaching is losing its symbionts, compromising the ability of the holobiont to cope with the physiological demand of photosynthesis. Two other pathways enriched by differentially expressed mRNAs were correlated with cell adhesion and the innate immune response, which comprises transcripts encoding focal adhesion proteins, cell adhesion molecules (CAMs) and primary immunodeficiency responsive components. Indeed, these results are corroborated by the work of Pinzon and coworkers who reported that the expression of immune-related genes would change during and after bleaching of a reef-building coral *Orbicella faveolata* ^13^.

**Table 1:** Top thirteen enriched KEGG pathways involving differentially expressed mRNAs in *P*. *caribaeorum*.

Pathway	Pathway ID	Number	Up/Down(Bleaching-vs-Healthy)	P value	P-corrected value
Photosynthesis	ko00195	20	0/20	5.367164e-11	1.406197e-08
Protein digestion and absorption	ko04974	34	7/27	1.039168e-08	1.361310e-06
Focal adhesion	ko04510	51	15/36	3.220733e-08	2.812773e-06
Ribosome	ko03010	41	16/25	4.539063e-08	2.973086e-06
Carbon fixation in photosynthetic organisms	ko00710	15	1/14	1.641190e-07	8.599836e-06
Photosynthesis - antenna proteins	ko00196	13	0/13	2.355030e-07	1.028363e-05
ECM-receptor interaction	ko04512	31	6/25	7.57104e-07	2.833732e-05
Cell adhesion molecules (CAMs)	ko04514	19	7/12	1.250847e-05	4.096524e-04
Amoebiasis	ko05146	26	8/18	1.733519e-05	5.046466e-04
Dilated cardiomyopathy	ko05414	36	5/31	0.0001710962	4.259353e-03
Hypertrophic cardiomyopathy (HCM)	ko05410	35	4/31	0.0001788278	4.259353e-03
Primary immunodeficiency	ko05340	9	0/9	0.0002033226	4.439210e-03
Pancreatic secretion	ko04972	17	8/9	0.0002288780	4.612772e-03

#### GO Annotation analysis of differentially expressed mRNAs in healthy and diseased (undergoing bleaching) *P*. *caribaeorum*

**Figure 10:**
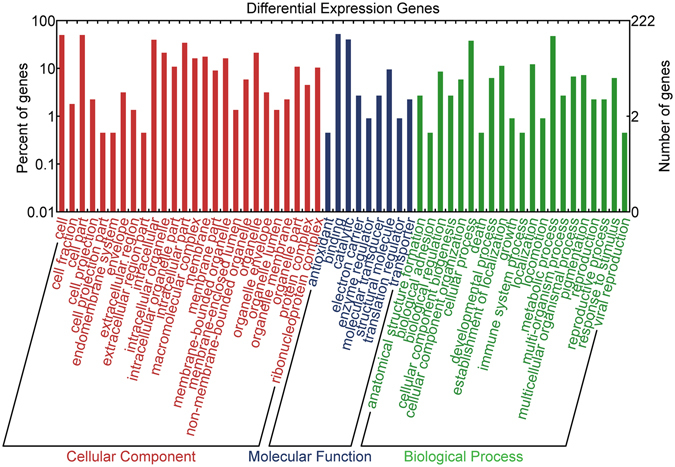
GO Annotation analysis of differentially expressed mRNAs in healthy and diseased (undergoing bleaching) *P*. *caribaeorum*.

Based on nr annotation, all of the differential expression mRNAs were mapped using the international standardized gene functional classification (GO) system. Concretely, Of the 1,119 most significant BLASTx hits against the nr database, a total of 222 differential expression mRNAs were annotated to at least one GO term, which could be categorized into 56 functional groups (Fig. 10). Among these, 134 (60.36%), 114 (51.35%) and 185 (83.33%) differential expression mRNAs were grouped into main categories comprising biological processes, cellular components and molecular functions, respectively. In each of the three main categories of GO classification, the terms “metabolic process”, “cell”, and “binding” account for the largest proportion. GO analysis revealed the functions of differential expression (both up-regulated and down-regulated) mRNAs in bleaching samples versus healthy samples. The functions of these mRNAs are probably related with many processes that are important in the bleaching response.

#### Predicted lncRNA-mRNA interactions in *P*. *caribaeorum* possibly implicated in coral bleaching

Previous studies from other researchers have shown that lncRNAs could play a regulatory role in diverse molecular pathways and processes of cells, in both healthy and disease states, being a determinant of the disease outcome in vertebrates, including humans. In the present study, a total of 200 significant differentially expressed lncRNAs (DELs) were identified by comparing the lncRNA expression level of healthy *P*. *caribaeorum* and individuals experiencing bleaching. As reported in the first part of this investigation regarding the prediction of lncRNA and their effect on the regulation of transcript translation and protein activity, we predicted target mRNAs for 29 lncRNAs in *P*. *caribaeorum*. However, none of them were DELs. This was not surprise for two main reasons: we adopted an extremely stringent strategy to screen the most reliable lncRNA-mRNA interactions, and mRNAs may not be necessarily be the principal target for lncRNAs, since even proteins may act as interplayers in lncRNA-mediated regulatory pathways^46^. In order to understand whether *P*. *caribaeorum* lncRNAs are implicated in coral bleaching and whether changes in the expression levels of lncRNAs correlate with the bleaching response, all of the DELs were compared to lncRNA sequences available in the NONCODE database, to search for probable conserved regions using a loose parameter (the E-value was adjusted to 10) by BLASTn. Interestingly, this analysis revealed numerous DELs that share short conserved regions (ranging from 19 bps to 50 bps) with lncRNAs deposited in NONCODE. Likewise, we adopted the same strategy that was mentioned in a previous section to search for probable target mRNAs. In total, 200 DELs were subjected to RNAplex to detect probable lncRNA-mRNA interactions among the 54,699 *P*. *caribaeorum* candidate mRNAs. By means of this strategy, one can verify that these mRNAs predictively interact with lncRNAs that tend to hybridize to conserved regions through almost-perfect complementary base pairing. These interacting partners were then annotated as candidate target mRNAs of DELs. Eventually, 89 lncRNA-mRNA interactions associated with the bleaching response in *P*. *caribaeorum* were identified, which involved interactions between 17 lncRNAs and 89 mRNAs. Annotation of the 89 target mRNAs by BLASTx against the nr database revealed that most DELs could act on the mRNAs encoding various enzymes correlated to DNA and RNA metabolism, including, for example, DNA-dependent RNA polymerase III, histone acetyltransferase and RNA-dependent DNA polymerase (Table 2). Additionally, some of these lncRNAs could also act on the mRNAs encoding regulatory proteins, such as transcription factor, splicing factor, THO complex, integrator complex subunit, elongator complex protein, and GTP-binding protein. Particularly, one *P*. *caribaeorum* lncRNA (ID: Unigene72046) is potentially able to interact with an mRNA encoding Ras-related protein. The Ras protein members have been reported to be ubiquitously expressed in all cell lineages and organs and generally they are involved in transmitting signals that would result in cell growth and division. It is well known that aberrant expression of Ras protein in human would be associated with proliferative diseases and over activity of ras signaling can lead to cancer^47^. Actually, genome annotation of the coral *A*. *digitifera* by Dunlap and coworkers has allowed disclosure of a large number of genes encoding putative Rab homologues of the Ras superfamily of proteins^48^. Differential expression analysis indicated that lncRNA Unigene72046 would be up-regulated in response to *P*. *caribaeorum* bleaching. Overexpression of lncRNA Unigene72046 might affect the transcriptional regulation of Ras-related protein, resulting in a disruption of intracellular signaling in *P*. *caribaeorum* individuals undergoing bleaching, that might culminate with a tentative recovery in cell growth, differentiation and survival.

**Table 2:** Detailed information of differentially expressed lncRNAs and predicted target mRNAs in *Palythoa caribaeorum*.

lncRNA ID	Target mRNAs number	Target mRNAs annotation information
Unigene72046^↑^	24	Aminotransferase; uncharacterized protein; trypsin-3-like; retinoic acid receptor; islet cell autoantigen; carbonic anhydrase; histone acetyltransferase; ranBP-type and C3HC4-type zinc finger-containing protein; epidermal growth factor receptor substrate; putative acyl-coenzyme A oxidase; ras-related protein Rab-24; probable UDP-sugar transporter protein; ubiquitin-like protein fubi and ribosomal protein; hypothetical protein; hypothetical protein*; NEDD8-conjugating enzyme; protease regulatory subunit; transmembrane protease;
Unigene69139^↑^	11	uncharacterized protein;stimulator of interferon genes protein; THO complex 1; complement factor B; transmembrane protein 181; charged multivesicular body protein; hypothetical protein; UDP-glucuronosyltransferase; elongator complex protein; histamine N-methyltransferase; integrator complex subunit;
Unigene57713^↓^	10	agglutinin biogenesis protein MshQ; hypothetical protein; predicted protein*; glutamate 5-kinase; methionine synthase; membrane protein metalloendopeptidase; unknown protein; MSHA biogenesis protein; GTP-binding protein;
Unigene7497^↓^	9	glycerol-3-phosphate acyltransferase; hypothetical protein; uncharacterized protein; N-acetylglucosaminyl-phosphatidylinositol de-N-acetylase; ubiquitin-conjugating enzyme; thiosulfate sulfurtransferase/rhodanese-like domain-containing protein; ubiquitin carboxyl-terminal hydrolase; RNA-directed DNA polymerase;
Unigene1657^↓^	6	enolase-like protein;predicted protein*; poly [ADP-ribose] polymerase isoform X2; poly(A) polymerase central domain protein; hypothetical protein; motile sperm domain-containing protein;
Unigene62981^↓^	6	cGMP-dependent 3′,5′-cyclic phosphodiesterase;chitinase 3-like; fibropellin-1-like; probable serine-O-acetyltransferase; phosphatidate cytidylyltransferase; uncharacterized protein;
Unigene83429^↓^	5	arginine/serine-rich protein PNISR isoform;protein kinase; splicing factor, arginine/serine-rich 18-like;
Unigene16589^↓^	4	uncharacterized protein; hypothetical protein; mannose-binding lectin associated serine protease; phytanoyl-CoA dioxygenase;
CL1738.Contig1^↑^	3	DNA-directed RNA polymerase III; uncharacterized protein*; predicted protein*;
CL7909.Contig1^↑^	3	predicted protein*; steroid 17-alpha-hydroxylase;
CL1253.Contig5^↑^	2	transcription factor MYTF; mitochondrial inner membrane protease subunit;
CL1417.Contig2^↑^	1	hypothetical protein;
CL4186.Contig1^↑^	1	uncharacterized protein;
Unigene55440^↓^	1	rab3 GTPase-activating protein catalytic subunit;
Unigene69919^↑^	1	glucose transporter;
Unigene70419^↑^	1	predicted protein*;
Unigene81587^↓^	1	predicted protein*;

^*^Represents protein uniquely annotated in cnidarian, i.e., Nematostella vectensis, Hydra vulgaris.

^↑^Represents up-regulated in Bleaching *P*. *caribaeorum* versus Healthy *P*. *caribaeorum*.

^↓^Represents down-regulated in Bleaching *P*. *caribaeorum* versus Healthy *P*. *caribaeorum*.

Intriguingly, one up-regulated *P*. *caribaeorum* lncRNA (ID: Unigene69139) was predicted to interact with mRNAs coding for immune-associated proteins, namely, the complement factor B and the stimulator of interferon genes protein (STING). Complement factor B is a component of an alternative pathway of complement activation, involved in the regulation of the innate immune response, and the stimulator of STING has been demonstrated to play an important role in innate immunity by inducing cells to produce immunomulators and, consequently, signalizing to control infection^49^. It is hypothesized here that the lncRNA (Unigene69139) would be able to posttranscriptionally regulate immune-related genes via direct interaction and that the up-regulation of this kind of lncRNA in *P*. *caribaeorum* undergoing bleaching, might severely impact the coral immune system in a tentative bid to control not only the lost beneficial symbionts, but also host susceptibility to pathogenic microbes. The KEGG analysis of differential expression mRNAs, presented in the previous section, indicates that components related to the immune response pathway were overall repressed in *P*. *variabilis* experiencing bleaching. Accordingly, Pinzon and collaborators also demonstrated that the coral immune response appears to be suppressed after a bleaching event^13^. It is well known and accepted that the immune system is a complex network that associated with numerous interconnected biological processes and pathways that play a crucial role in maintaining the balance of an organism, particularly in fighting disease. Furthermore, in the case of corals, immune system interplayers also participate in the colonization of self (species-specific)-beneficial microscopic dwellers to the detriment of infectious microbes. Our findings indicated that overexpression of lncRNAs that potentially regulate immune-related mRNAs could contribute to the host immunosuppression in response to coral bleaching.

## Discussion

The rapid development of sequencing technologies has allowed the discovery of tens of thousands of lncRNAs in recent years^4, 50^. They were initially thought of as being “transcriptional noise”, due to the incompetence of encoding proteins^4, 51^. However, cumulative studies now indicate that lncRNAs comprise a novel class of significant biological regulators, which has been implicated in a range of biological processes, including development and differentiation. Additionally, lncRNAs have emerged as another class of cellular components that influences the disease outcome. However, most studies about lncRNAs were until now restricted to few established model organisms^52–54^. For instance, the NONCODE database integrates sets of data with ncRNAs of 16 species, including human, mouse, cow, rat, chicken, fruitfly, zebrafish, nematode (*C*. *elegans*) and yeast^55^. Commonly, lncRNAs possess a low level of structural and sequence conservation among species^46^. Hence, the fact that most lncRNAs diverge considerably among species, and that they have been found in a limited number of model organisms, causes some difficulties in straightforwardly identifying lncRNAs from unusual non-model organism by means of comparative homology sequence searches and multi-alignments. Moreover, with the exception of their functions, lncRNAs are paradoxically very similar to mRNAs in several structural aspects, such as the overall organization of precursor structures and regions of base-pairing complementarity. Compared to mRNAs, the majority of mature lncRNAs are generated by the same histone modifications; that is, the same RNA polymerase II transcriptional machinery and they also are accordingly polyadenylated^45, 56^, suggesting that *a priori* lncRNAs are indistinguishable from functional mRNAs. However, this is exactly the reason why a relatively large number of lncRNA sequences can be retrieved when one conducts RNA sequencing merely based on the RNA ‘Poly (A)’ library. In the present work, aiming at the identification of lncRNAs in the transcriptomes of two anthozoan species, we initiated our survey by taking data from next-generation RNA sequencing performed in accordance with our previously published studies^28, 29^, but now including an adapted filtering pipeline step for analytical identification of subsets of lncRNAs. In this way, we were able to predict a large number of lncRNAs in the transcriptomes of two species of zoanthids one of which undergoing bleaching. The number of predicted lncRNAs in these species of cnidarians exceeded that which was initially expected. This could be attributable to several differences in our experimental designs and from those of other researchers. For instance, Wang and collaborators^29^ predicted lncRNAs in *Panax ginseng* from EST sequences, and not from deep RNA sequencing. In a work by Gaiti and colleagues^28^, they combined genome data to filter for lncRNAs, creating potentially more chances to eliminate not only false-positive lncRNA candidates, but also true positives. Indeed, lncRNAs in some ways are regarded as a moniker until they are better characterized; a small percentage of transcripts originally reported as lncRNAs have later been found to have the capacity to encode new (usually small) proteins^50, 57^. We believe that our *in silico* prediction of lncRNA repertoires from the transcriptomes of understudied living organisms could in the future be further optimized, along with specific and constant improvement in sequencing data handling and bioinformatics, particularly concerning to ncRNA analysis.

In the light of lncRNA functions, besides their well-characterized effects as regulators of transcription, a small proportion of lncRNAs have been known for their roles as posttranscriptional regulators, involved in pre-mRNA splicing, RNA editing, mRNA decay, translation and abrogation of miRNA-induced repression^6, 58^. However, functional characterization of identified lncRNAs has been great challenge so far. On the one hand, effective experimental approaches for validating lncRNA functions cannot deal with the increasing amount of transcriptional data generated. On the other hand, bioinformatics methods utilized to predict lncRNA functions are still in the early stage. Certainly, a large number of studies have successfully elucidated the functional role of some lncRNAs in the molecular biology of cancer and chronic diseases^59–62^. Wang and his collaborators, for example, revealed that approximately 40% of lncRNAs, named mRNA-like ncRNAs, are the precursors of microRNAs in *P*. *ginseng* that mature to produce multi-function–associated elements responsive to jasmonate (–a plant signaling molecule)^29^. Ren and coworkers^63^ investigated the target genes regulated by lncRNAs in the process of skin pigmentation and development based on the deep RNA-sequencing data of dark and white goats.

Several studies have suggested that a great number of lncRNAs exert their effects via base-paring with complementary DNAs and RNAs^6, 58, 64^. In fact, purely sequence-based methods like BLAST and FASTA, which search for long stretches of perfect complementarity between two queried RNAs, have been used for detecting probable RNA-DNA/RNA interactions. For instance, Szcześniak and collaborators predicted a large number of lncRNA-RNA interactions in human transcriptome using a similarity-search method^58^. As an alternative, He *et al*. developed a tool named LongTarget to predict lncRNA-DNA interactions via base-pairing analysis^65^. In our present study, we initially investigated the content of lncRNAs and the pattern of expression in the transcriptomes of two marine basal organisms – the anthozoans *P*. *variabilis* and *P*. *caribaeorum*, parentally related to the first metazoans that arose in the oceans more than 500 million years ago. Since *P*. *caribaeorum*, in the coral reefs that it inhabits, is the first species to display the symptoms of bleaching, we predicted a possible network of lncRNAs interactions in this zoanthid species under two physiological conditions: healthy tissue and colonial individuals undergoing bleaching. As shown here, we found that a few *P*. *caribaeorum* lncRNAs shared relatively short but highly conserved regions with known lncRNAs derived from higher organisms. Based on this finding, we reasoned that these conserved regions could be associated with *P*. *caribaeorum* lncRNA target-interaction and regulatory functions. Concurrently, the lncRNA-mRNA interactions were predicted using RNAplex and indicated that the majority of the putative hybridization sites were restricted to conserved lncRNA segments (regions). Therefore, we proposed an alternative strategy to potentially screen more lncRNAs-mRNAs interactions in *P*. *caribaeorum* transcriptomes, and hypothesized that such lncRNA interactions are implicated in posttranscriptional regulation in *P*. *caribaeorum* according to differences in metabolic status.

The fact that coral bleaching, a sort of ‘disease outbreak’ of anthozoans, has increased in frequency in recent decades has pushed many researchers to investigate the mechanism(s) of coral bleaching response and holobiont collapse. A number of biological processes and pathways, as well as DGEs related to bleaching have been revealed by means of deep RNA sequencing of distinct unrelated coral species. Nevertheless, the implication for lncRNAs in corals, in general, and for anthozoan transcriptomes, of coral bleaching in particular were not taken into account prior to the present study.

Altogether, interesting data emerged from our analysis: firstly, the repertoires of lncRNAs in *P*. *variabilis* and *P*. *caribaeorum* are quantitatively and qualitatively more homogeneous than among organisms of distinct phyla; secondly, cross-interaction of lncRNAs of *P*. *caribaeorum* with a group of mRNAs from a phylogenetically close species of cnidarians, like the starlet sea anemone, suggested conservation with respect to the target-drive function of lncRNAs. Another remarkable discovery is the variation in the expression level of certain lncRNAs in the transcriptomes of healthy *P*. *caribaeorum* versus colonial individuals undergoing bleaching, as well as identification of their potential target mRNAs, which also displayed variable levels of expression and are probably implicated in the underlying molecular response to bleaching. As can be observed here, our analysis suggests that some up-regulated DELs might be involved in the posttranscriptional regulation of mRNAs encoding two important groups of protein effectors: one related to immune responses and another to Ras intracellular signaling. Acting in concert in the bleaching response of *P*. *caribaeorum*, these groups of multi-effector polypeptides (and their mRNA precursors) are recruited and modulated in a tentative bid to mitigate pathogenic microbial assault and control infection and promote tissue remodeling to maintain symbionts and the functionality of the holobiont. It is known that allorecognition, xenorecognition and restriction (killing reaction) are inherent abilities of anthozoans, allowing them to thrive in harsh aquatic environments. In fact, the mechanism of underlying interaction between host and symbiont relies on distinction by organisms and cells of self from non-self, which in turn depends on proteins and signaling pathways concerned with the innate immune response and molecular patterns of cell recognition and adhesion^66, 67^. Furthermore, there is an increasing number of examples showing that cross-kingdom RNA *trans*-regulation is a process that triggers gene silencing and regulation mediated by small RNA and double-stranded long RNA that translocate bi-directionally from the host to the partner symbiont/pathogen^68^. In this context, one can conceive that lncRNAs might act as additional interplayers in the array of molecular dispositive to discriminate self (symbionts) from non-self (pathogens) and regulate the residence of beneficial microorganisms that are detrimental to pathogens. To our knowledge, our findings disclose for the first time the presence of lncRNAs in the transcriptomes of two species of zoanthids, and pave the way for a new perspective on the molecular mechanisms of coral bleaching, as demonstrated in healthy and diseased (undergoing bleaching) *P*. *caribaeorum* by transcriptome analysis.

## Electronic supplementary material

Supplementary information
