# Supplementary material for: Identification of long non-coding RNAs in two anthozoan species and their possible implications for coral bleaching
Source: Sci Rep. 2017 Jul 13;7:5333. doi: 10.1038/s41598-017-02561-y (PMC5509713; doi:10.1038/s41598-017-02561-y)
Supplement: Supplementary file 1 — Supplementary information [file 41598_2017_2561_MOESM1_ESM.pdf]

Supplementary Information

Identification of long non-coding RNAs in two anthozoan species and their possible implication for coral bleaching

Chen Huang[1], Jean-Étienne RL Morlighem[2,3], Simon Ming Yuen Lee[1*], Qiwen Liao[1], Carlos Daniel Perez[4], Paula Braga Gomes[5], Min Guo[1], Gandhi Rádis-Baptista[2*], Cheuk-Wing Li[1*]

[1]State Key Laboratory of Quality Research in Chinese Medicine and Institute of Chinese Medical Sciences, University of Macau, Macau, China.
[2]Northeast Biotechnology Network (RENORBIO), Post-graduation program in Biotechnology, Federal University of Ceará, Brazil.
[3]Laboratory of Biochemistry and Biotechnology, Institute for Marine Sciences, Federal University of Ceará, Brazil.
[4]Centro Acadêmico de Vitoria, Universidade Federal de Pernambuco, Vitória de Santo Antão, Brazil
[5]Departamento de Biologia, Universidade Federal Rural de Pernambuco, Recife, Brazil

Authors for correspondence:
Cheuk-Wing Li,
State Key Laboratory of Quality Research in Chinese Medicine, Institute of Chinese Medical Sciences, University of Macau, Macau, China.
E-mail: cheukwli@umac.mo

Simon Ming Yuen Lee,
State Key Laboratory of Quality Research in Chinese Medicine, Institute of Chinese Medical Sciences, University of Macau, Macau, China.
E-mail: simonlee@umac.mo

Gandhi Rádis-Baptista,
Laboratory of Biochemistry and Biotechnology, Institute for Marine Sciences, Federal University of Ceará, Av. da Abolição, 3207, 60165-081 Fortaleza, CE, Brazil.
E-mail: gandhi.radis@ufc.br

Supplementary Figures

Figure S1. Sequencing quality report of RNA-seq of *P.variabilis* by FastQC. A) Kmer content of raw reads from fq1 file of *P.variabilis*. **B)** Per base sequence quality of raw reads from fq1 file of *P.variabilis*. **C)** Per base sequence content of raw reads from fq1 file of *P.variabilis*. **D)** Kmer content of raw reads from fq2 file of *P.variabilis*. **E)** Per base sequence quality of raw reads from fq2 file of *P.variabilis*. **F)** Per base sequence content of raw reads from fq2 file of *P.variabilis*. **G)** Kmer content of clean reads from fq1 file of *P.variabilis*. **H)** Per base sequence quality of clean reads from fq1 file of *P.variabilis*. **I)** Per base sequence content of clean reads from fq1 file of *P.variabilis*. **J)** Kmer content of clean reads from fq2 file of *P.variabilis*. **K)** Per base sequence quality of clean reads from fq2 file of *P.variabilis*. **L)** Per base sequence content of clean reads from fq2 file of *P.variabilis*.

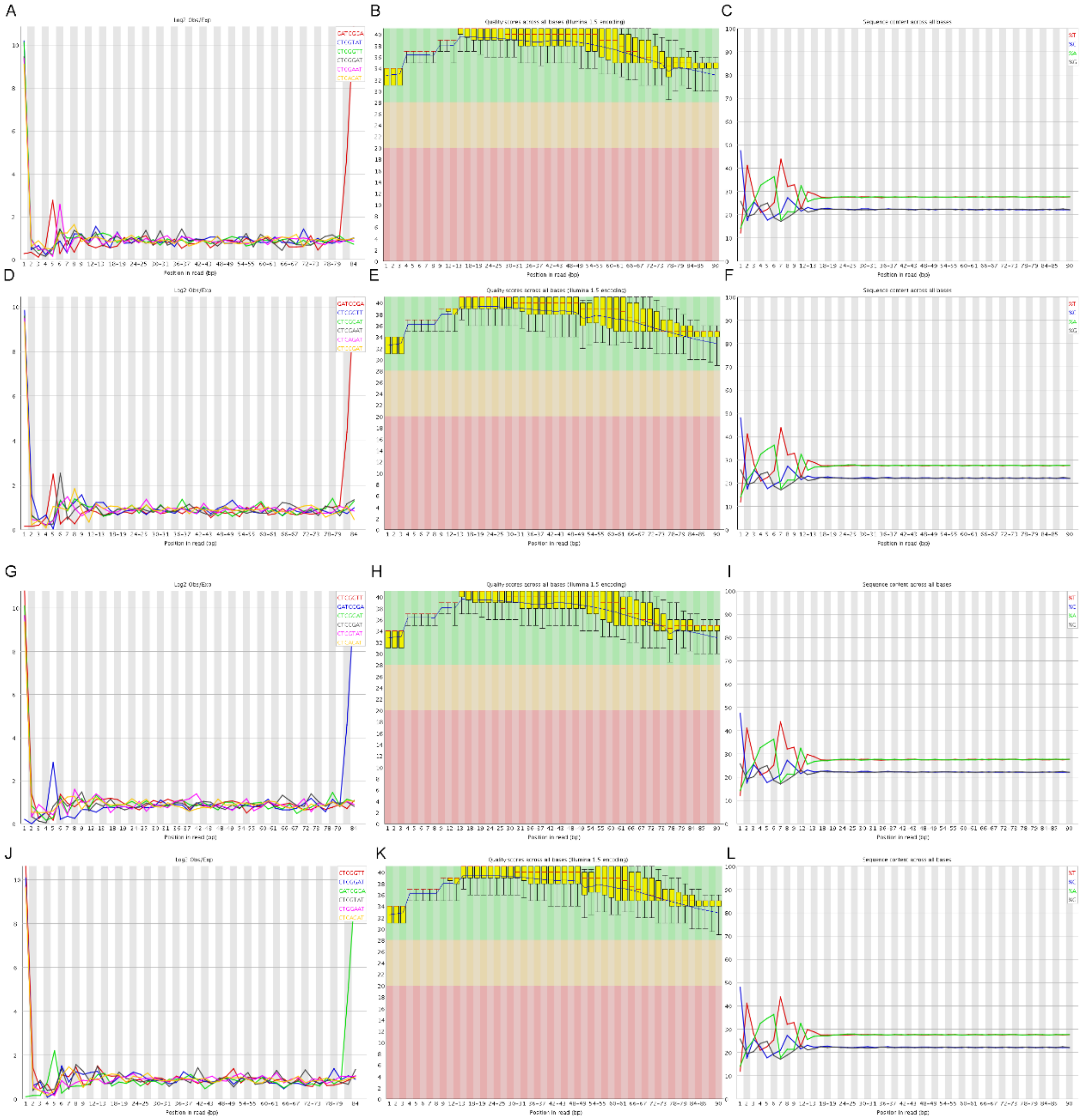

Figure S2. Sequencing quality report of RNA-seq of healthy *P. caribaeorum* by FastQC. A) Kmer content of raw reads from fq1 file of healthy *P. caribaeorum*. **B)** Per base sequence quality of raw reads from fq1 file of healthy *P. caribaeorum*. **C)** Per base sequence content of raw reads from fq1 file of healthy *P. caribaeorum*. **D)** Kmer content of raw reads from fq2 file of healthy *P. caribaeorum*. **E)** Per base sequence quality of raw reads from fq2 file of healthy *P. caribaeorum*. **F)** Per base sequence content of raw reads from fq2 file of healthy *P. caribaeorum*. **G)** Kmer content of clean reads from fq1 file of healthy *P. caribaeorum*. **H)** Per base sequence quality of clean reads from fq1 file of healthy *P. caribaeorum*. **I)** Per base sequence content of clean reads from fq1 file of healthy *P. caribaeorum*. **J)** Kmer content of clean reads from fq2 file of healthy *P. caribaeorum*. **K)** Per base sequence quality of clean reads from fq2 file of healthy *P. caribaeorum*. **L)** Per base sequence content of clean reads from fq2 file of healthy *P. caribaeorum*.

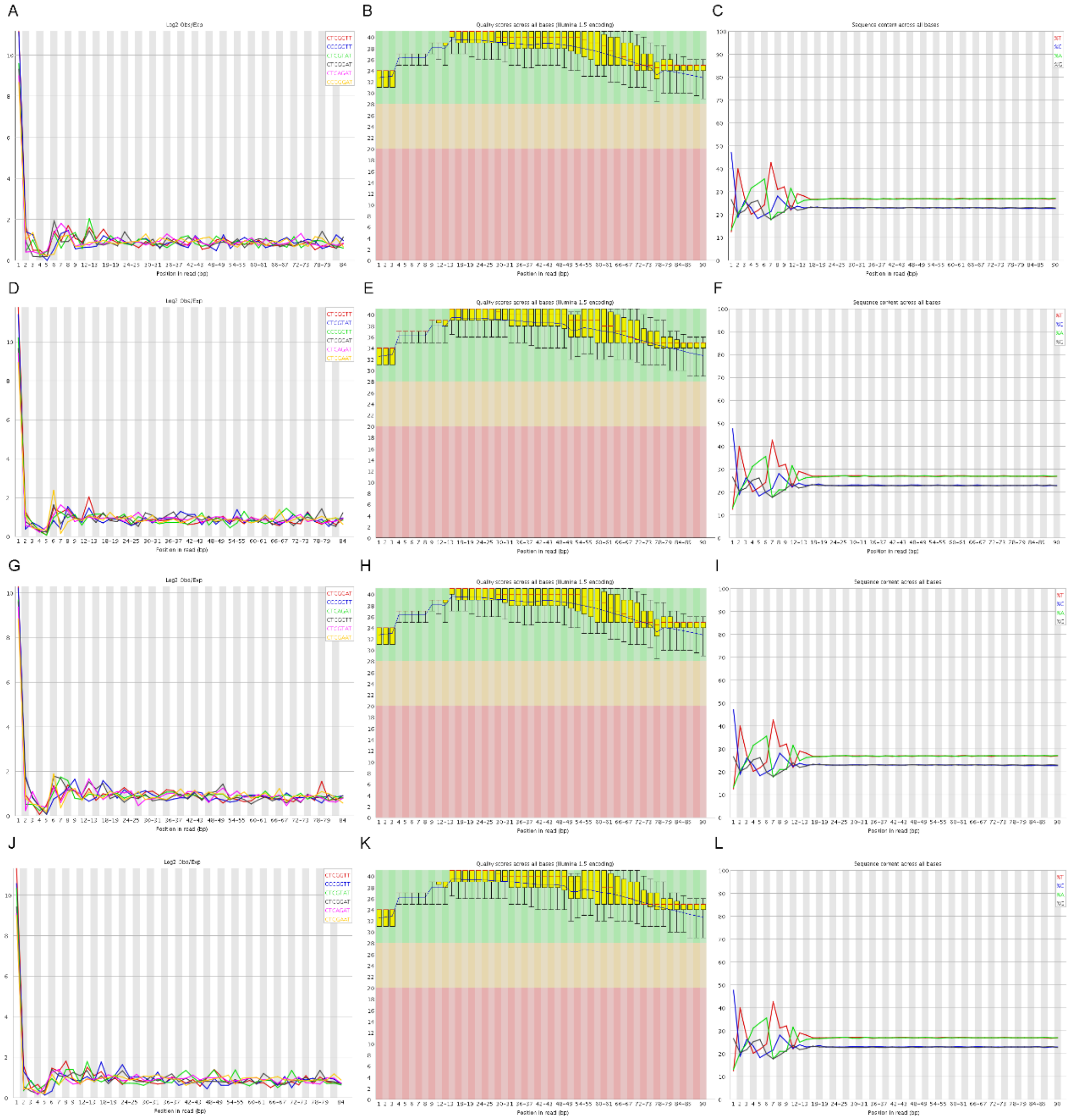

Figure S3. Sequencing quality report of RNA-seq of *P. caribaeorum* undergoing bleaching by FastQC. A) Kmer content of raw reads from fq1 file of *P. caribaeorum* undergoing bleaching. **B)** Per base sequence quality of raw reads from fq1 file of *P. caribaeorum* undergoing bleaching. **C)** Per base sequence content of raw reads from fq1 file of *P. caribaeorum* undergoing bleaching. **D)** Kmer content of raw reads from fq2 file of *P. caribaeorum* undergoing bleaching. **E)** Per base sequence quality of raw reads from fq2 file of *P. caribaeorum* undergoing bleaching. **F)** Per base sequence content of raw reads from fq2 file of *P. caribaeorum* undergoing bleaching. **G)** Kmer content of clean reads from fq1 file of *P. caribaeorum* undergoing bleaching. **H)** Per base sequence quality of clean reads from fq1 file of *P. caribaeorum* undergoing bleaching. **I)** Per base sequence content of clean reads from fq1 file of *P. caribaeorum* undergoing bleaching. **J)** Kmer content of clean reads from fq2 file of *P. caribaeorum* undergoing bleaching. **K)** Per base sequence quality of clean reads from fq2 file of *P. caribaeorum* undergoing bleaching. **L)** Per base sequence content of clean reads from fq2 file of *P. caribaeorum* undergoing bleaching.

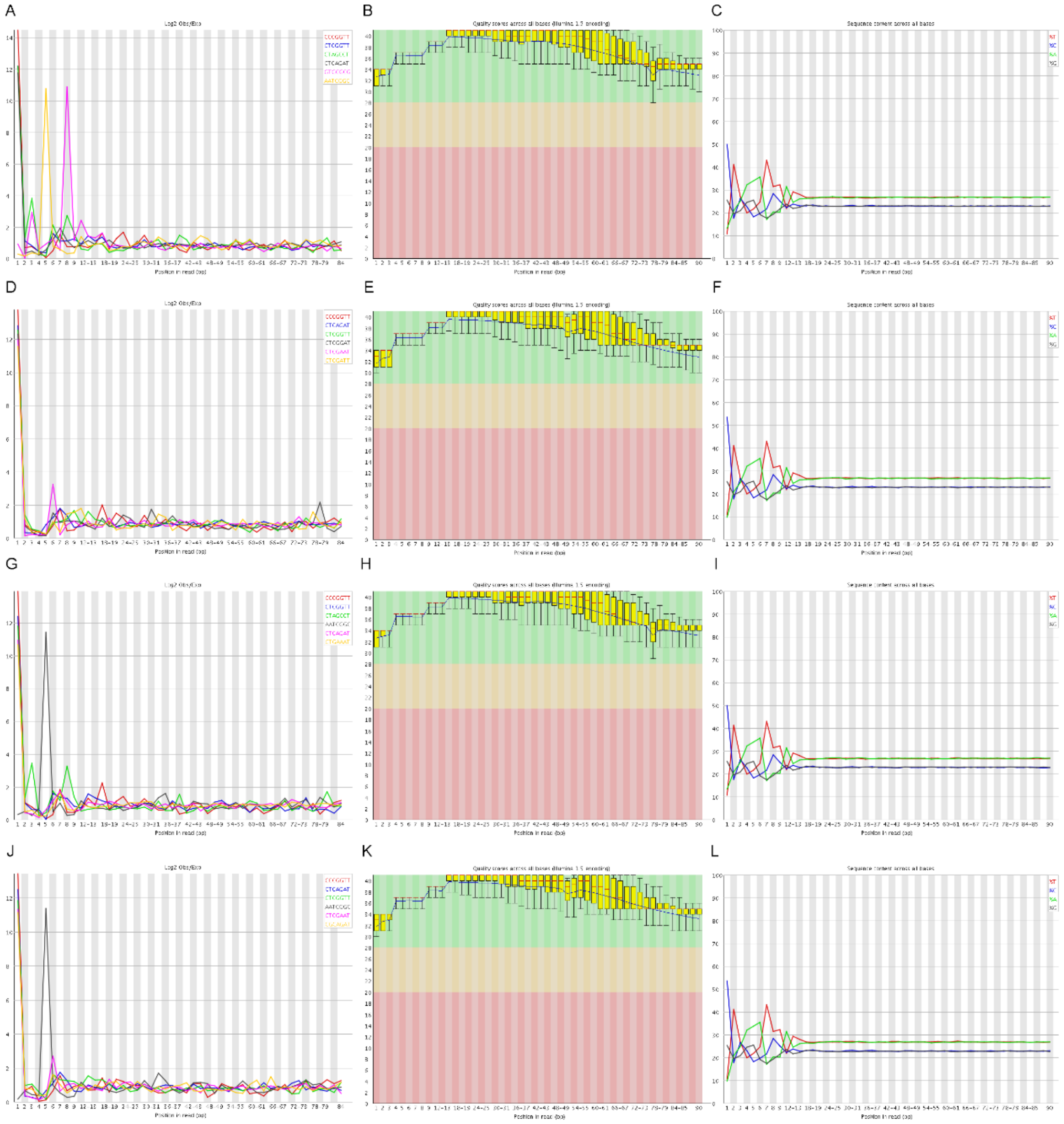

Figure S4. Species Distribution of putative lncRNAs in *P. variabilis* and *P. caribaeorum* determined by the best sequence hits. The lncRNA-like sequences of *P. variabilis* and *P. caribaeorum* were annotated by comparing with the lncRNA sequences in NONCODE v3.0 database using BLASTN, respectively (E-value≤1.0E-3). Part A –*P. caribaeorum*. Part-B –*P. variabilis*.

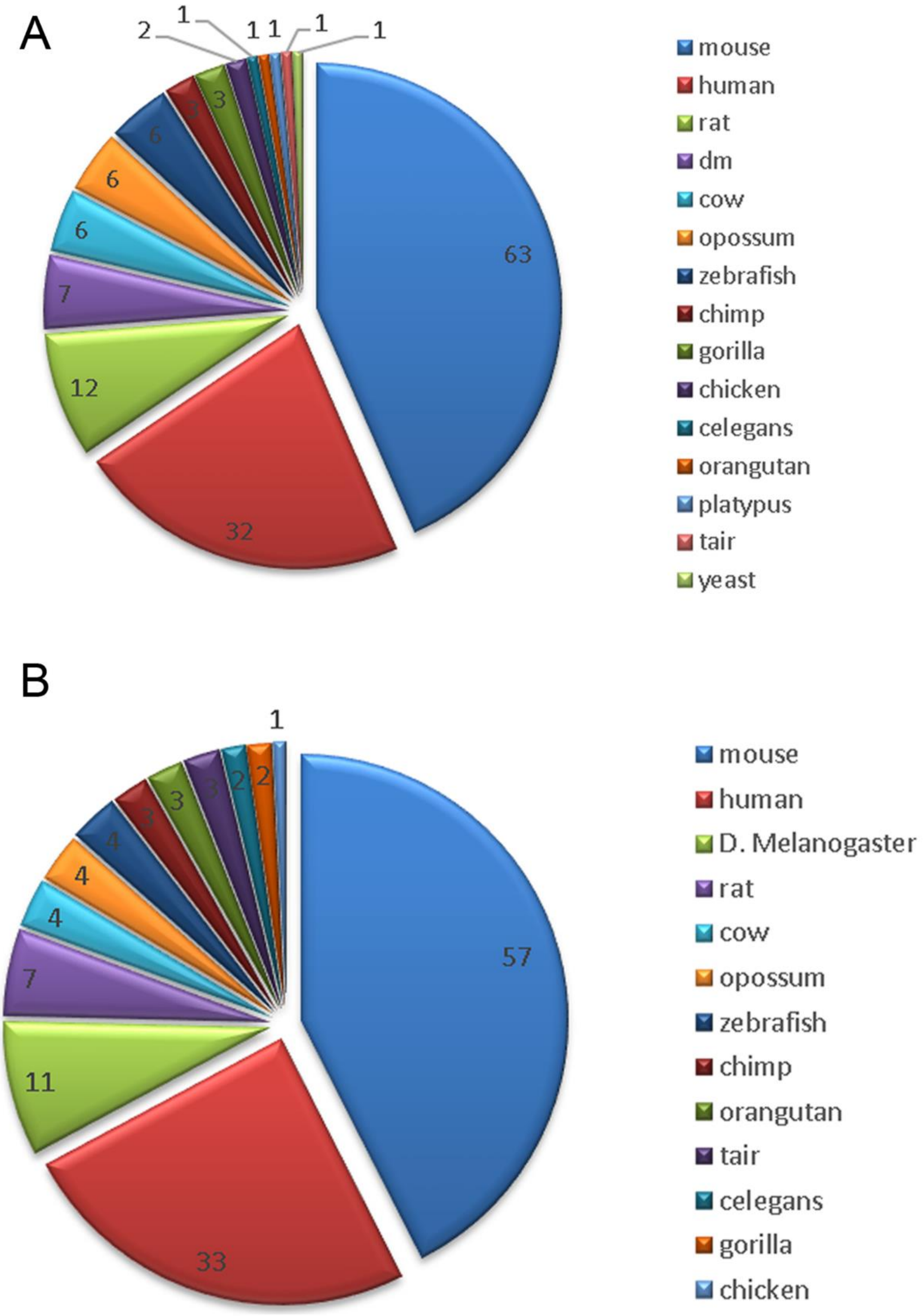

Figure S5 Comparison of *P. caribaeorum* lncRNA (CL4490.Contig2) to the lncRNAs derived from diverse model species. A) Sketch map of localization of conserved regions of *P. caribaeorum* lncRNA (CL4490.Contig2) in the lncRNAs (NONDREG203605, NONHSAG287712, NONPTRG204298, and NONMMUG317856 in NONCODE v3.0 database) of diverse model species: *Palythoa*, *Zebrafish*, *Human*, *Chimpanzee* and *Mouse*. **B)** Multiple alignment of the region of conservation of the *P. caribaeorum* lncRNA with compared lncRNAs in *Zebrafish*, *Human*, *Chimpanzee* and *Mouse*.

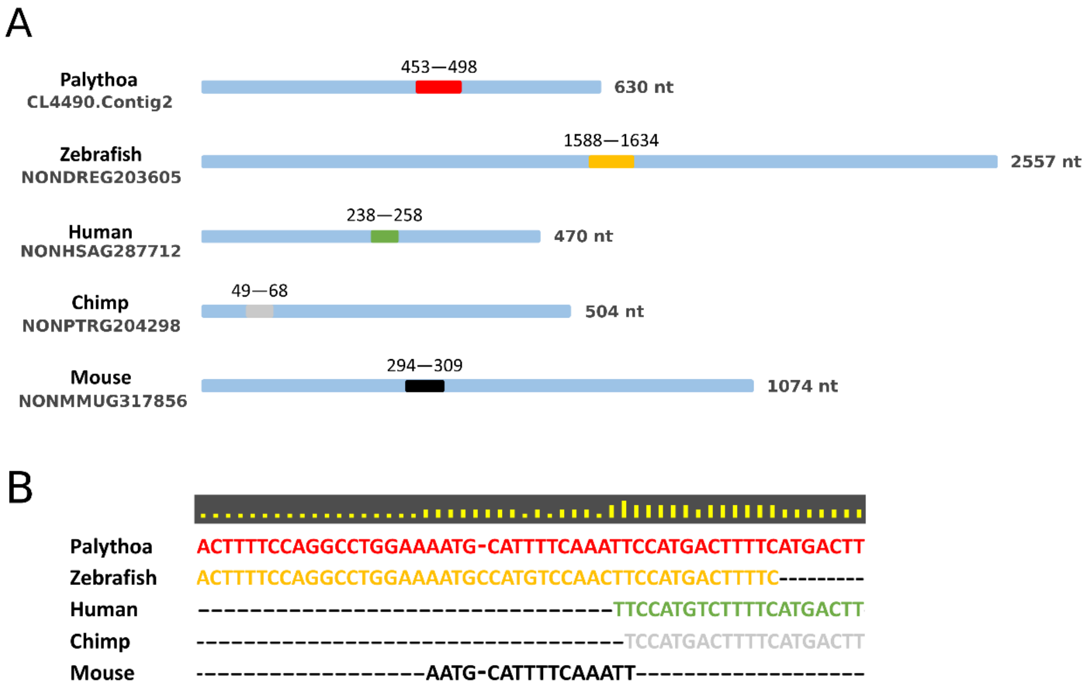

Figure S6 Interaction pattern of lncRNA with target mRNA based on conserved region proposed in *P. caribaeorum*. A) The secondary structures of Human lncRNA (NONHSAG31852, NONCODE v3.0 database), conserved region was highlighted in red. **B)** The secondary structures of *Palythoa* lncRNA (Unigene14915), conserved region was highlighted in red. Sequence alignment of the human and coral conserved regions was displayed in upper red elliptical frame. **C)** The secondary structures of target mRNA which was predicted to interact with lncRNA (Unigene14915). Interaction region was extracted from RNAplex RNA-RNA interaction prediction (free energy

required for hybridization predicted in RNAplex: -35.70). The interaction region in mRNA (Unigene65833, BLAST against nr annotation revealed that this mRNA may encode transcription factor, with accession No.: XP_007473380, XP_003214327, XP_011490426, XP_007112019, XP_012906212, XP_008103493, XP_007112018, XP_010815684) was highlighted in red. Details of sequences for hybridization were displayed in lower red elliptical frame. The secondary structures for all RNAs were predicted on RNAfold webserver.

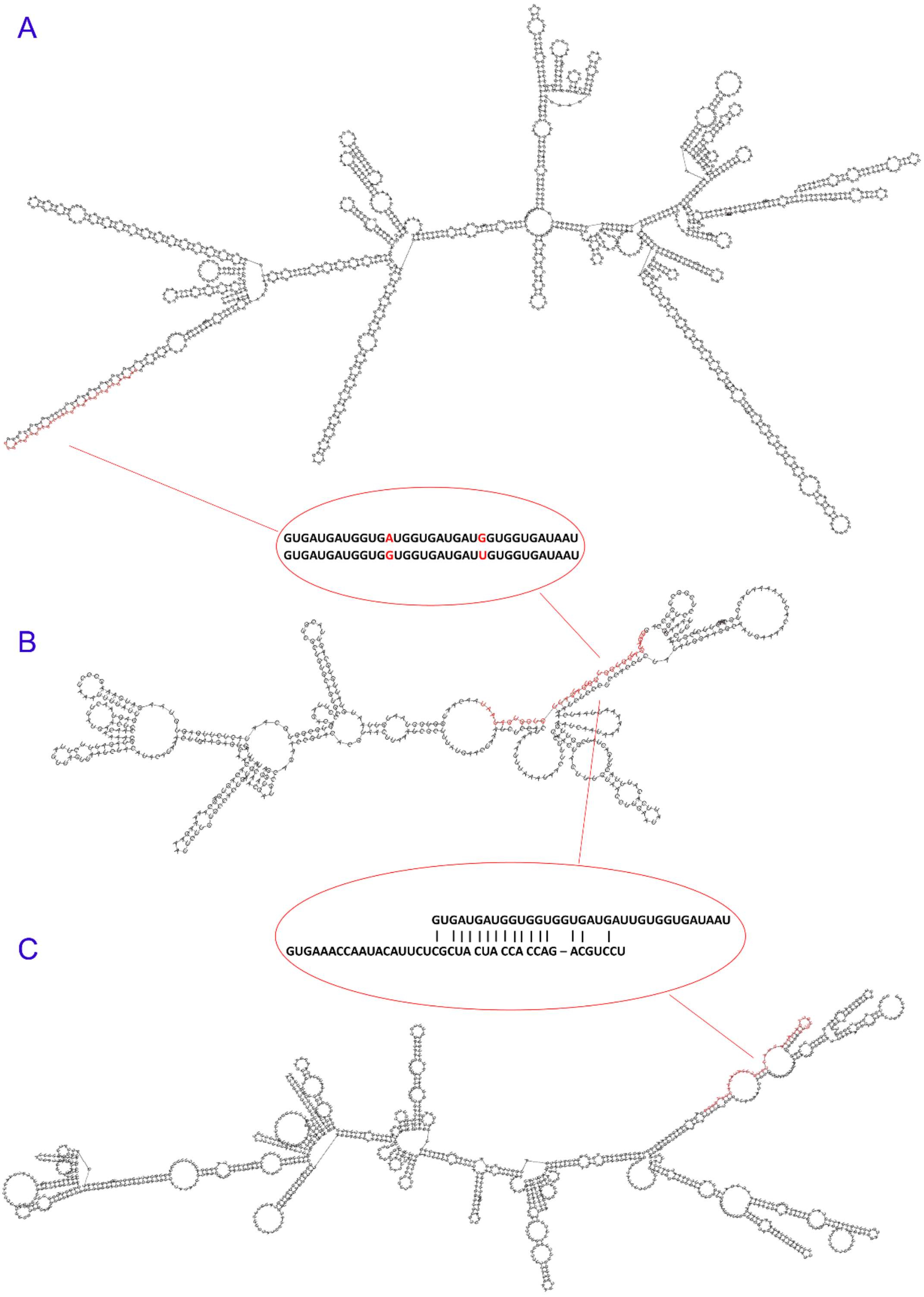

Supplementary Tables

Table S1 Summary of lncRNA-mRNA interaction predicted based on RNAplex in *P. caribaeorum*.

P. caribaeorum **lncRNA ID**	**mRNAs with hybridization sites located in conserved region**	**mRNAs with high complementary base-pairing(>=80%)**	**mRNAs with free energy lower than -20**
Unigene89595_All	50255	112	104
Unigene14915_All	52454	45	45
Unigene70263_All	36190	43	40
Unigene72836_All	47774	27	26
Unigene40263_All	39649	22	22
CL9785.Contig2_All	50292	19	16
Unigene63045_All	7934	24	16
Unigene102630_All	49840	15	14
Unigene53506_All	42199	15	14
CL1040.Contig5_All	36809	14	14
CL1040.Contig6_All	36172	14	13
Unigene2105_All	53311	13	11
Unigene39741_All	27626	19	10
Unigene49834_All	41533	11	9
Unigene61949_All	49943	7	7
CL1040.Contig1_All	36713	6	6
CL9996.Contig2_All	49508	9	5
Unigene60195_All	21384	5	5
Unigene29690_All	37905	5	4
Unigene9030_All	6080	6	4
Unigene56911_All	53026	4	3
CL7909.Contig1_All	292	4	3
Unigene23949_All	9379	6	2
Unigene64887_All	941	2	2
Unigene2220_All	564	3	2
Unigene3670_All	198	3	2
Unigene35955_All	47263	5	1
Unigene22615_All	1658	2	1
CL10350.Contig2_All	19	1	1
CL6141.Contig4_All	50013	0	0
Unigene9191_All	29227	3	0
Unigene56469_All	20109	4	0
Unigene36322_All	8259	0	0
CL1102.Contig1_All	4798	0	0
Unigene88144_All	4449	0	0
Unigene22011_All	1395	0	0
Unigene22395_All	1205	0	0
CL1491.Contig1_All	1106	0	0
Unigene26574_All	941	0	0

Unigene24008_All	536	0	0
Unigene76351_All	444	0	0
Unigene23102_All	443	0	0
Unigene12276_All	341	0	0
Unigene93514_All	329	0	0
Unigene67286_All	323	0	0
Unigene49840_All	318	0	0
Unigene26118_All	308	0	0
Unigene50184_All	291	0	0
Unigene41692_All	286	0	0
Unigene8984_All	249	0	0
Unigene85491_All	209	0	0
Unigene2194_All	188	0	0
Unigene37819_All	170	0	0
Unigene34830_All	154	1	0
Unigene84770_All	131	0	0
Unigene90867_All	93	0	0
Unigene94275_All	66	0	0
Unigene66881_All	53	1	0
Unigene39492_All	39	0	0
CL10350.Contig1_All	36	0	0
CL723.Contig2_All	25	0	0
CL2237.Contig1_All	17	0	0
CL11050.Contig1_All	14	0	0
Unigene39380_All	12	0	0
Unigene44074_All	11	0	0
CL642.Contig2_All	10	0	0
Unigene22480_All	7	0	0
Unigene32986_All	7	0	0
Unigene65174_All	7	0	0
Unigene61140_All	6	0	0
Unigene50589_All	4	0	0
CL9362.Contig1_All	4	0	0
Unigene87777_All	4	0	0
Unigene92982_All	4	0	0
Unigene44595_All	3	0	0
CL10736.Contig4_All	3	0	0
Unigene8108_All	3	0	0
Unigene102120_All	2	0	0
Unigene104851_All	2	0	0
Unigene22452_All	2	0	0
Unigene9322_All	2	0	0
Unigene14_All	2	0	0
Unigene56906_All	2	0	0
Unigene43485_All	2	0	0
Unigene10478_All	2	0	0
Unigene9509_All	2	0	0
CL8218.Contig3_All	2	0	0
Unigene36593_All	2	0	0
Unigene16462_All	1	0	0

Unigene60170_All	1	0	0
Unigene71355_All	1	0	0
Unigene73002_All	1	0	0
Unigene37956_All	1	0	0
CL5008.Contig1_All	1	0	0
CL4621.Contig2_All	1	0	0
Unigene25828_All	1	0	0
Unigene63062_All	1	0	0
Unigene50030_All	1	0	0
CL9.Contig3_All	1	0	0
Unigene86657_All	1	0	0
Unigene50075_All	1	0	0
Unigene80323_All	1	0	0
CL2084.Contig1_All	1	0	0
CL8569.Contig2_All	1	0	0
Unigene36319_All	1	0	0
CL5008.Contig2_All	1	0	0
Unigene73318_All	1	0	0
Unigene30249_All	1	0	0
Unigene22880_All	1	0	0
Unigene20717_All	1	0	0
Unigene43899_All	1	0	0
Unigene9459_All	1	0	0
CL4392.Contig4_All	1	0	0
CL2719.Contig2_All	1	0	0
Unigene64790_All	1	0	0
Unigene56653_All	1	0	0
CL4490.Contig2_All	1	0	0
Unigene76828_All	1	0	0
Unigene70256_All	1	0	0
Unigene10244_All	1	0	0
Unigene9745_All	1	0	0
Unigene27300_All	1	0	0
Unigene98246_All	1	0	0
Unigene87955_All	1	0	0
Unigene2266_All	1	0	0
CL5834.Contig2_All	1	0	0
Unigene82196_All	1	0	0
Unigene26491_All	1	0	0
CL8569.Contig1_All	1	0	0
Unigene82707_All	1	0	0
CL4621.Contig1_All	0	0	0
Unigene10186_All	0	0	0
Unigene24155_All	0	0	0
Unigene31177_All	0	0	0
Unigene3654_All	0	0	0
Unigene42022_All	0	0	0
Unigene49751_All	0	0	0
Unigene49832_All	0	0	0
Unigene57101_All	0	0	0

Unigene71054_All	0	0	0
Unigene71734_All	0	0	0
Unigene74999_All	0	0	0
Unigene75592_All	0	0	0
Unigene80203_All	0	0	0
Unigene91279_All	0	0	0

Table S2 Summary of target mRNA annotation of 29 DELs in *P. caribaeorum*.

lncRNA ID	Interacted mRNA	mRNA Annotation
CL10350.Contig2_All	Unigene98670_All	transcriptase
CL1040.Contig1_All	Unigene30352_All	uncharacterized protein
CL1040.Contig1_All	Unigene45920_All	SCO-spondin-like
CL1040.Contig1_All	Unigene55508_All	hypothetical protein
CL1040.Contig1_All	Unigene59190_All	MFS transporter
CL1040.Contig1_All	Unigene8463_All	hypothetical protein
CL1040.Contig1_All	Unigene88175_All	beta-hydroxylase
CL1040.Contig5_All	CL3674.Contig2_All	Plasma membrane iron permease
CL1040.Contig5_All	Unigene1669_All	AT-rich interactive domain-containing protein
CL1040.Contig5_All	Unigene21311_All	Unannotated
CL1040.Contig5_All	Unigene29083_All	oxidoreductase
CL1040.Contig5_All	Unigene30352_All	uncharacterized protein
CL1040.Contig5_All	Unigene41966_All	hydroxylase/oxygenase
CL1040.Contig5_All	Unigene43654_All	Unannotated
CL1040.Contig5_All	Unigene45920_All	SCO-spondin-like
CL1040.Contig5_All	Unigene46088_All	Ankyrin repeat family protein
CL1040.Contig5_All	Unigene55508_All	hypothetical protein
CL1040.Contig5_All	Unigene59190_All	MFS transporter
CL1040.Contig5_All	Unigene61123_All	copine-4
CL1040.Contig5_All	Unigene65307_All	ASCH domain-containing protein
CL1040.Contig5_All	Unigene88175_All	beta-hydroxylase
CL1040.Contig6_All	Unigene21045_All	EGF and pentraxin domain-containing protein
CL1040.Contig6_All	Unigene21311_All	Unannotated
CL1040.Contig6_All	Unigene29516_All	DOM3 exoribonuclease
CL1040.Contig6_All	Unigene43654_All	Unannotated
CL1040.Contig6_All	Unigene45920_All	SCO-spondin-like
CL1040.Contig6_All	Unigene53451_All	beta-galactosidase-like
CL1040.Contig6_All	Unigene53871_All	metallo-beta-lactamase domain-containing protein
CL1040.Contig6_All	Unigene55508_All	hypothetical protein
CL1040.Contig6_All	Unigene61818_All	PKSN polyketide synthase
CL1040.Contig6_All	Unigene65307_All	ASCH domain-containing protein
CL1040.Contig6_All	Unigene7691_All	hypothetical protein
CL1040.Contig6_All	Unigene7979_All	hypothetical protein
CL1040.Contig6_All	Unigene88175_All	beta-hydroxylase
CL7909.Contig1_All	CL2716.Contig3_All	predicted protein
CL7909.Contig1_All	CL2716.Contig4_All	17-alpha-hydroxylase

CL7909.Contig1_All	CL2716.Contig5_All	predicted protein
CL9785.Contig2_All	CL2200.Contig2_All	Unannotated
CL9785.Contig2_All	CL6342.Contig2_All	gamma-glutamyltranspeptidase
CL9785.Contig2_All	CL6342.Contig3_All	gamma-glutamyltranspeptidase
CL9785.Contig2_All	CL77.Contig3_All	low-density lipoprotein receptor-related protein
CL9785.Contig2_All	CL77.Contig4_All	low-density lipoprotein receptor-related protein
CL9785.Contig2_All	Unigene11638_All	ABC transporter ATP-binding protein
CL9785.Contig2_All	Unigene12015_All	nuclear factor NF-kappa-B
CL9785.Contig2_All	Unigene15108_All	Unannotated
CL9785.Contig2_All	Unigene19278_All	strombine dehydrogenase
CL9785.Contig2_All	Unigene21400_All	regulatory ankyrin repeat subunit B-like isoform X2
CL9785.Contig2_All	Unigene28365_All	selenoprotein SelM2
CL9785.Contig2_All	Unigene34632_All	uncharacterized protein
CL9785.Contig2_All	Unigene34709_All	Brefeldin A-inhibited guanine nucleotide-exchange protein
CL9785.Contig2_All	Unigene38690_All	predicted protein
CL9785.Contig2_All	Unigene40168_All	DEAD/DEAH box helicase
CL9785.Contig2_All	Unigene48176_All	Unannotated
CL9996.Contig2_All	CL1636.Contig2_All	Unannotated
CL9996.Contig2_All	CL6680.Contig2_All	centrosomal protein of 19 kDa
CL9996.Contig2_All	Unigene25859_All	acyl-coenzyme A thioesterase
CL9996.Contig2_All	Unigene59116_All	neuroligin-4, X-linked isoform X2
CL9996.Contig2_All	Unigene6467_All	leucine rich repeat-containing protein
Unigene102630_All	CL10751.Contig2_All	ATP-dependent RNA helicase
Unigene102630_All	CL5978.Contig1_All	3-phosphoglycerate dehydrogenase
Unigene102630_All	CL5978.Contig2_All	3-phosphoglycerate dehydrogenase
Unigene102630_All	CL8390.Contig1_All	hypothetical protein
Unigene102630_All	CL8390.Contig2_All	predicted protein
Unigene102630_All	CL8390.Contig3_All	hypothetical protein
Unigene102630_All	CL8390.Contig4_All	predicted protein
Unigene102630_All	Unigene12184_All	zinc finger MYM-type protein 3
Unigene102630_All	Unigene13965_All	small RNA degrading nuclease
Unigene102630_All	Unigene19492_All	CST complex subunit TEN1
Unigene102630_All	Unigene35606_All	unknown protein
Unigene102630_All	Unigene35656_All	Enhancer of rudimentary like protein
Unigene102630_All	Unigene42578_All	hypothetical protein
Unigene102630_All	Unigene44349_All	Unannotated
Unigene14915_All	CL1193.Contig2_All	serine hydrolase
Unigene14915_All	CL139.Contig1_All	Unannotated
Unigene14915_All	CL139.Contig2_All	Unannotated
Unigene14915_All	CL4384.Contig1_All	regulator of nonsense transcripts
Unigene14915_All	CL531.Contig2_All	phosphatidylethanolamine-binding protein
Unigene14915_All	CL531.Contig3_All	phosphatidylethanolamine-binding protein
Unigene14915_All	CL5366.Contig3_All	serine-arginine protein 55 isoform X7
Unigene14915_All	CL5627.Contig3_All	senescence-associated protein
Unigene14915_All	CL6122.Contig1_All	predicted protein
Unigene14915_All	CL6726.Contig2_All	sodium-dependent multivitamin transporter
Unigene14915_All	CL7262.Contig1_All	protein boule-like
Unigene14915_All	CL7289.Contig1_All	lipoxygenase
Unigene14915_All	CL7289.Contig2_All	lipoxygenase

Unigene14915_All	CL7425.Contig1_All	zinc transporter 7
Unigene14915_All	CL8285.Contig1_All	hypothetical protein
Unigene14915_All	Unigene14878_All	5' AMP activated protein kinase
Unigene14915_All	Unigene20290_All	cytochrome c oxidase subunit
Unigene14915_All	Unigene20643_All	calcium-binding protein
Unigene14915_All	Unigene222_All	predicted protein
Unigene14915_All	Unigene24745_All	voltage-gated sodium channel
Unigene14915_All	Unigene27869_All	Multisite-specific tRNA:(cytosine-C(5))-methyltransferase
Unigene14915_All	Unigene29045_All	ATP-dependent DNA helicase
Unigene14915_All	Unigene29285_All	uncharacterized protein
Unigene14915_All	Unigene33387_All	glycosyl hydrolase
Unigene14915_All	Unigene34046_All	alpha-1,6-mannosylglycoprotein 6-beta-N-acetylglucosaminyltransferase A
Unigene14915_All	Unigene38136_All	MYND finger domain-containing protein
Unigene14915_All	Unigene38282_All	sulfate transporter
Unigene14915_All	Unigene39647_All	RNA-directed DNA polymerase
Unigene14915_All	Unigene42064_All	GNAT family N-acetyltransferase
Unigene14915_All	Unigene47931_All	lipoprotein receptor-related protein
Unigene14915_All	Unigene48466_All	hypothetical protein
Unigene14915_All	Unigene49140_All	uncharacterized protein
Unigene14915_All	Unigene49312_All	potassium channel
Unigene14915_All	Unigene52317_All	protein ELC-like
Unigene14915_All	Unigene5272_All	Unannotated
Unigene14915_All	Unigene53522_All	Unannotated
Unigene14915_All	Unigene53679_All	membrane protein
Unigene14915_All	Unigene54284_All	40S ribosomal protein
Unigene14915_All	Unigene59365_All	amino acid permease
Unigene14915_All	Unigene65652_All	39S ribosomal protein
Unigene14915_All	Unigene65833_All	transcription factor
Unigene14915_All	Unigene66111_All	Krueppel-like factor 6 isoform X2
Unigene14915_All	Unigene73090_All	Tenascin-X
Unigene14915_All	Unigene87385_All	metallophosphoesterase
Unigene14915_All	sodium-dependent	multivitamin transporter isoform X3
Unigene2105_All	CL1591.Contig2_All	ATP-binding cassette protein C4-like
Unigene2105_All	CL2983.Contig1_All	tight junction protein
Unigene2105_All	CL2983.Contig2_All	tight junction protein ZO-1 isoform
Unigene2105_All	CL3469.Contig1_All	acetylcholine receptor subunit
Unigene2105_All	Unigene11307_All	predicted protein
Unigene2105_All	Unigene38919_All	ubiquitin thiolesterase
Unigene2105_All	Unigene41420_All	Xaa-Pro aminopeptidase
Unigene2105_All	Unigene59316_All	unknown protein
Unigene2105_All	Unigene62479_All	Na channel protein
Unigene2105_All	Unigene67843_All	unknown protein
Unigene2105_All	Unigene7486_All	transcription factor 3C polypeptide
Unigene2220_All	CL2572.Contig2_All	acyl-CoA-binding protein
Unigene2220_All	Unigene61766_All	RNA-directed DNA polymerase
Unigene22615_All	Unigene9021_All	Unannotated
Unigene23949_All	CL5109.Contig2_All	Unannotated
Unigene23949_All	Unigene43049_All	Unannotated

Unigene29690_All	CL3197.Contig2_All	PEP-CTERM domain protein
Unigene29690_All	CL4559.Contig1_All	anaphase-promoting complex subunit 6
Unigene29690_All	Unigene33586_All	hypothetical protein
Unigene29690_All	Unigene5996_All	myelin transcription factor
Unigene35955_All	CL9649.Contig1_All	polyadenylate-binding protein
Unigene3670_All	CL5066.Contig2_All	endonuclease-reverse transcriptase
Unigene3670_All	Unigene21558_All	reverse transcriptase
Unigene39741_All	CL1393.Contig1_All	predicted protein
Unigene39741_All	CL1393.Contig3_All	hypothetical protein
Unigene39741_All	CL149.Contig1_All	titin-like
Unigene39741_All	CL149.Contig2_All	predicted protein
Unigene39741_All	Unigene22169_All	ATP-dependent DNA helicase
Unigene39741_All	Unigene34466_All	Golgi-associated PDZ and coiled-coil motif-containing protein
Unigene39741_All	Unigene49141_All	ceramide-1-phosphate transfer
Unigene39741_All	Unigene52733_All	isocitrate dehydrogenase [NAD] subunit
Unigene39741_All	Unigene54339_All	rRNA-processing protein FCF1-like protein
Unigene39741_All	Unigene65966_All	vacuolar protein sorting-associated protein
Unigene40263_All	CL2327.Contig2_All	hypothetical protein
Unigene40263_All	CL2883.Contig1_All	T-complex protein 1 subunit
Unigene40263_All	CL2883.Contig2_All	T-complex protein 1 subunit
Unigene40263_All	CL2924.Contig5_All	hypothetical protein
Unigene40263_All	CL4384.Contig1_All	regulator of nonsense transcripts
Unigene40263_All	CL531.Contig2_All	phosphatidylethanolamine-binding protein
Unigene40263_All	Unigene18545_All	hypothetical protein
Unigene40263_All	Unigene45723_All	NHL repeat containing protein
Unigene40263_All	Unigene48296_All	flavin oxidoreductase
Unigene40263_All	Unigene49084_All	hypothetical protein
Unigene40263_All	Unigene49098_All	dentin sialophosphoprotein-like isoform X1
Unigene40263_All	Unigene52818_All	hypothetical protein
Unigene40263_All	Unigene54402_All	phosphoribosylformylglycinamidine cyclo-ligase
Unigene40263_All	Unigene55086_All	LMBR1 domain-containing protein
Unigene40263_All	Unigene55538_All	CAMK family protein kinase
Unigene40263_All	Unigene59365_All	amino acid permease
Unigene40263_All	Unigene62283_All	phosphoacetylglucosamine mutase
Unigene40263_All	Unigene62363_All	zinc transporter
Unigene40263_All	Unigene66055_All	serine/arginine-rich splicing factor
Unigene40263_All	Unigene66164_All	protein mig c
Unigene40263_All	Unigene80006_All	hypothetical protein
Unigene40263_All	Unigene87385_All	metallophosphoesterase
Unigene49834_All	CL2593.Contig1_All	uncharacterized protein
Unigene49834_All	CL2895.Contig2_All	serine/threonine-protein kinase MARK1
Unigene49834_All	CL2895.Contig3_All	serine/threonine-protein kinase
Unigene49834_All	CL2895.Contig4_All	serine/threonine-protein kinase MARK1
Unigene49834_All	CL2895.Contig5_All	MAP/microtubule affinity-regulating kinase
Unigene49834_All	Unigene2609_All	tubulin polymerization-promoting protein
Unigene49834_All	Unigene43528_All	cullin-associated NEDD8-dissociated protein
Unigene49834_All	Unigene47977_All	low affinity copper uptake protein
Unigene49834_All	Unigene60754_All	Unannotated
Unigene53506_All	Unigene17780_All	ADPribosylglycohydrolas

Unigene53506_All	Unigene18874_All	hypothetical protein
Unigene53506_All	Unigene19980_All	arginine N-methyltransferase
Unigene53506_All	Unigene21350_All	hypothetical protein
Unigene53506_All	Unigene23498_All	lymphoid-specific helicase isoform X1
Unigene53506_All	Unigene32147_All	tetratricopeptide repeat-containing protein
Unigene53506_All	Unigene32368_All	hypothetical protein
Unigene53506_All	Unigene40541_All	Unannotated
Unigene53506_All	Unigene41423_All	transposase
Unigene53506_All	Unigene44911_All	aminotransferase
Unigene53506_All	Unigene5084_All	Bardet-Biedl syndrome
Unigene53506_All	Unigene63760_All	hypothetical protein
Unigene53506_All	Unigene7677_All	histidine ammonia-lyase isoform
Unigene53506_All	Unigene8709_All	GPN-loop GTPase 3-like
Unigene56911_All	Unigene18046_All	membrane protein
Unigene56911_All	Unigene40638_All	calcium-binding protein
Unigene56911_All	Unigene51874_All	protein disulfide-isomerase
Unigene60195_All	Unigene10965_All	soluble calcium-activated nucleotidase
Unigene60195_All	Unigene26442_All	uncharacterized protein
Unigene60195_All	Unigene48554_All	Unannotated
Unigene60195_All	Unigene52108_All	biliverdin reductase A
Unigene60195_All	Unigene73090_All	Tenascin-X
Unigene61949_All	CL2363.Contig1_All	translation initiation factor eIF-2B subunit
Unigene61949_All	CL736.Contig1_All	2-acylglycerol O-acyltransferase
Unigene61949_All	CL9532.Contig1_All	tetratricopeptide repeat protein
Unigene61949_All	Unigene19097_All	translation initiation factor
Unigene61949_All	Unigene25151_All	Clathrin interactor 1
Unigene61949_All	Unigene29646_All	alpha-ketoglutarate-dependent dioxygenase
Unigene61949_All	Unigene39647_All	RNA-directed DNA polymerase
Unigene63045_All	CL149.Contig10_All	predicted protein
Unigene63045_All	CL149.Contig1_All	titin-like
Unigene63045_All	CL149.Contig2_All	predicted protein
Unigene63045_All	CL149.Contig8_All	predicted protein
Unigene63045_All	CL2262.Contig7_All	Unannotated
Unigene63045_All	CL4531.Contig1_All	CCR4-NOT transcription complex subunit
Unigene63045_All	CL4531.Contig2_All	CCR4-NOT transcription complex subunit
Unigene63045_All	CL661.Contig4_All	succinate dehydrogenase assembly factor
Unigene63045_All	CL7643.Contig2_All	SCF E3 ubiquitin ligase complex F-box protein
Unigene63045_All	Unigene24611_All	uncharacterized protein
Unigene63045_All	Unigene34106_All	RNA-directed DNA polymerase
Unigene63045_All	Unigene40498_All	predicted protein
Unigene63045_All	Unigene49356_All	pol-like protein
Unigene63045_All	Unigene54767_All	transient receptor potential cation channel subfamily A
Unigene63045_All	Unigene65480_All	RNA polymerase II elongation factor
Unigene63045_All	Unigene8021_All	SPRY domain-containing protein
Unigene64887_All	CL6529.Contig1_All	Unannotated
Unigene64887_All	Unigene81338_All	Unannotated
Unigene70263_All	CL1243.Contig1_All	host cell factor
Unigene70263_All	CL1243.Contig2_All	host cell factor
Unigene70263_All	CL1243.Contig3_All	host cell factor

Unigene70263_All	CL1773.Contig3_All	hemicentin-1 isoform
Unigene70263_All	CL1773.Contig5_All	hemicentin-2 isoform
Unigene70263_All	CL2009.Contig1_All	hypothetical protein
Unigene70263_All	CL2856.Contig1_All	endonuclease-reverse transcriptase
Unigene70263_All	CL3084.Contig2_All	RING finger and CHY zinc finger domain-containing protein
Unigene70263_All	CL452.Contig2_All	predicted protein
Unigene70263_All	CL4665.Contig1_All	Unannotated
Unigene70263_All	CL4840.Contig1_All	BRCA2 and CDKN1A-interacting protein
Unigene70263_All	CL4840.Contig2_All	uncharacterized protein
Unigene70263_All	CL625.Contig2_All	rabankyrin-5 isoform
Unigene70263_All	CL6784.Contig1_All	alpha-1,3-mannosyl-glycoprotein 4-beta-N-acetylglucosaminyltransferase
Unigene70263_All	CL7118.Contig2_All	hypothetical protein
Unigene70263_All	CL8374.Contig1_All	hypothetical protein
Unigene70263_All	CL8374.Contig2_All	uncharacterized protein
Unigene70263_All	CL8425.Contig1_All	4-substituted benzoates-glutamate ligase
Unigene70263_All	SPRY	domain-containing protein
Unigene70263_All	Unigene11424_All	succinate dehydrogenase cytochrome b560 subunit
Unigene70263_All	Unigene13101_All	Protein kinase C epsilon type
Unigene70263_All	Unigene1804_All	Steroid 17-alpha-hydroxylase
Unigene70263_All	Unigene21183_All	glycohydrolase
Unigene70263_All	Unigene22169_All	ATP-dependent DNA helicase
Unigene70263_All	Unigene27762_All	zinc finger protein
Unigene70263_All	Unigene34189_All	kinesin family member 3C isoform
Unigene70263_All	Unigene40101_All	DNA replication complex GINS protein
Unigene70263_All	Unigene40498_All	predicted protein
Unigene70263_All	Unigene48396_All	WD repeat domain phosphoinositide-interacting protein
Unigene70263_All	Unigene49293_All	palmitoyltransferase
Unigene70263_All	Unigene49356_All	pol-like protein
Unigene70263_All	Unigene58079_All	Unannotated
Unigene70263_All	Unigene58936_All	CAP-Gly domain-containing linker protein
Unigene70263_All	Unigene61766_All	RNA-directed DNA polymerase
Unigene70263_All	Unigene65615_All	N-alpha-acetyltransferase
Unigene70263_All	Unigene69925_All	hypothetical protein
Unigene70263_All	Unigene8021_All	SPRY domain-containing protein
Unigene70263_All	Unigene8051_All	voltage-gated sodium channel
Unigene70263_All	Unigene80640_All	hypothetical protein
Unigene70263_All	Unigene8711_All	CUE domain-containing protein
Unigene72836_All	CL1026.Contig2_All	protein IMPACT isoform
Unigene72836_All	CL1296.Contig2_All	hypothetical protein
Unigene72836_All	CL1627.Contig3_All	hypothetical protein
Unigene72836_All	CL1858.Contig3_All	protein spinster homolog 1-like isoform
Unigene72836_All	CL1858.Contig4_All	protein spinster homolog 1-like isoform
Unigene72836_All	CL578.Contig1_All	sphingosine-1-phosphate phosphatase
Unigene72836_All	CL8544.Contig4_All	sodium/myo-inositol cotransporter
Unigene72836_All	CL9822.Contig2_All	predicted protein
Unigene72836_All	Unigene13140_All	predicted protein
Unigene72836_All	Unigene25481_All	FH1/FH2 domain-containing protein
Unigene72836_All	Unigene36298_All	ion transporter

Unigene72836_All	Unigene38745_All	predicted protein
Unigene72836_All	Unigene39669_All	Membrane associated eicosanoid/glutathione metabolism-like domain protein
Unigene72836_All	Unigene42597_All	testis-expressed sequence 2 protein
Unigene72836_All	Unigene45783_All	tetratricopeptide TPR_2 repeat protein
Unigene72836_All	Unigene47478_All	phosphatidylserine synthase
Unigene72836_All	Unigene50803_All	DNA helicase MCM8
Unigene72836_All	Unigene52409_All	Kunitz/Bovine pancreatic trypsin inhibitor domain protein
Unigene72836_All	Unigene54193_All	guanylate cyclase soluble subunit
Unigene72836_All	Unigene566_All	helicase with zinc finger domain
Unigene72836_All	Unigene59493_All	hypothetical protein
Unigene72836_All	Unigene62698_All	phytanoyl-CoA dioxygenase domain-containing protein
Unigene72836_All	Unigene62813_All	gastrula zinc finger protein
Unigene72836_All	Unigene67293_All	copper/zinc superoxide dismutase
Unigene72836_All	Unigene90891_All	Growth factor receptor-bound protein
Unigene72836_All	Unigene9829_All	protein spinster homolog 1-like isoform
Unigene89595_All	CL10387.Contig1_All	nuclear factor 1 C-type-like isoform
Unigene89595_All	CL10695.Contig1_All	4-hydroxybenzoyl-CoA thioesterase
Unigene89595_All	CL10913.Contig1_All	Unannotated
Unigene89595_All	CL10956.Contig11_All	truncated actin-4
Unigene89595_All	CL10956.Contig6_All	muscle-specific actin-like
Unigene89595_All	CL1225.Contig1_All	ecto-NOX disulfide-thiol exchanger
Unigene89595_All	CL1236.Contig1_All	FGFR1 oncogene partner 2 isoform
Unigene89595_All	CL1782.Contig2_All	uncharacterized protein
Unigene89595_All	CL1782.Contig5_All	uncharacterized protein
Unigene89595_All	CL1782.Contig6_All	uncharacterized protein
Unigene89595_All	CL1787.Contig1_All	hypothetical protein
Unigene89595_All	CL208.Contig1_All	tripartite motif-containing protein 45 isoform
Unigene89595_All	CL208.Contig2_All	filamin-A isoform
Unigene89595_All	CL208.Contig3_All	predicted protein
Unigene89595_All	CL2387.Contig4_All	lissencephaly-1 homolog
Unigene89595_All	CL267.Contig10_All	zinc finger MIZ domain-containing protein
Unigene89595_All	CL267.Contig11_All	zinc finger MIZ domain-containing protein
Unigene89595_All	CL267.Contig12_All	zinc finger MIZ domain-containing protein
Unigene89595_All	CL267.Contig13_All	zinc finger MIZ domain-containing protein
Unigene89595_All	CL267.Contig14_All	zinc finger MIZ domain-containing protein
Unigene89595_All	CL267.Contig15_All	zinc finger MIZ domain-containing protein
Unigene89595_All	CL267.Contig16_All	zinc finger MIZ domain-containing protein
Unigene89595_All	CL267.Contig17_All	zinc finger MIZ domain-containing protein
Unigene89595_All	CL267.Contig18_All	zinc finger MIZ domain-containing protein
Unigene89595_All	CL267.Contig19_All	zinc finger MIZ domain-containing protein 1-like
Unigene89595_All	CL267.Contig20_All	zinc finger MIZ domain-containing protein
Unigene89595_All	CL267.Contig21_All	zinc finger MIZ domain-containing protein
Unigene89595_All	CL267.Contig22_All	zinc finger MIZ domain-containing protein
Unigene89595_All	CL267.Contig23_All	Zinc finger MIZ domain-containing protein
Unigene89595_All	CL267.Contig24_All	zinc finger MIZ domain-containing protein
Unigene89595_All	CL267.Contig26_All	zinc finger MIZ domain-containing protein
Unigene89595_All	CL267.Contig27_All	zinc finger MIZ domain-containing protein
Unigene89595_All	CL267.Contig4_All	zinc finger MIZ domain-containing protein

Unigene89595_All	CL267.Contig5_All	zinc finger MIZ domain-containing protein
Unigene89595_All	CL267.Contig6_All	zinc finger MIZ domain-containing protein
Unigene89595_All	CL267.Contig7_All	zinc finger MIZ domain-containing protein
Unigene89595_All	CL267.Contig8_All	zinc finger MIZ domain-containing protein
Unigene89595_All	CL267.Contig9_All	zinc finger MIZ domain-containing protein
Unigene89595_All	CL2693.Contig1_All	NB-ARC domain containing protein
Unigene89595_All	CL3123.Contig2_All	nicotinamidase
Unigene89595_All	CL3123.Contig3_All	nicotinamidase
Unigene89595_All	CL3123.Contig5_All	nicotinamidase
Unigene89595_All	CL3123.Contig8_All	nicotinamidase-like
Unigene89595_All	CL3630.Contig1_All	hypothetical protein
Unigene89595_All	CL3630.Contig2_All	hypothetical protein
Unigene89595_All	CL3853.Contig1_All	hypothetical protein
Unigene89595_All	CL4079.Contig1_All	ras-related protein
Unigene89595_All	CL4079.Contig2_All	ras-related protein Rab-13
Unigene89595_All	CL4079.Contig3_All	ras-related protein Rab-13
Unigene89595_All	CL4223.Contig1_All	hypothetical protein
Unigene89595_All	CL556.Contig1_All	7-methylguanosine phosphate-specific 5'-nucleotidase A-like isoform
Unigene89595_All	CL556.Contig2_All	7-methylguanosine phosphate-specific 5'-nucleotidase A-like
Unigene89595_All	CL736.Contig1_All	2-acylglycerol O-acyltransferase
Unigene89595_All	CL7650.Contig1_All	predicted protein
Unigene89595_All	CL773.Contig7_All	calcium binding EGF domain protein
Unigene89595_All	CL786.Contig6_All	uncharacterized protein
Unigene89595_All	CL90.Contig2_All	mRNA-decapping enzyme
Unigene89595_All	CL9717.Contig1_All	uncharacterized protein
Unigene89595_All	CL9717.Contig2_All	uncharacterized protein
Unigene89595_All	CL982.Contig3_All	Crystal Structure Of Engineered Protein
Unigene89595_All	Unigene100976_All	metallophosphoesterase
Unigene89595_All	Unigene11443_All	protein BTG1-like
Unigene89595_All	Unigene13472_All	nudC domain-containing protein
Unigene89595_All	Unigene13535_All	thioredoxin
Unigene89595_All	Unigene13673_All	uncharacterized protein
Unigene89595_All	Unigene16098_All	transcription elongation regulator
Unigene89595_All	Unigene16937_All	hypothetical protein
Unigene89595_All	Unigene18453_All	complement component C3 precursor
Unigene89595_All	Unigene18878_All	cytochrome B5
Unigene89595_All	Unigene21644_All	nuclear transcription factor
Unigene89595_All	Unigene25782_All	unknown protein
Unigene89595_All	Unigene27665_All	selenocysteine-specific elongation factor isoform
Unigene89595_All	Unigene27978_All	chromosome-associated kinesin KIF4
Unigene89595_All	Unigene28589_All	disintegrin and metalloproteinase domain-containing protein
Unigene89595_All	Unigene33215_All	14 kDa subunit splicing factor 3b
Unigene89595_All	Unigene34189_All	kinesin family member 3C isoform
Unigene89595_All	Unigene34787_All	loricrin-like isoform
Unigene89595_All	Unigene36063_All	unconventional myosin-IXb-like isoform
Unigene89595_All	Unigene38515_All	predicted protein
Unigene89595_All	Unigene42938_All	predicted protein
Unigene89595_All	Unigene4482_All	hypothetical protein

Unigene89595_All	Unigene45123_All	tektin-1 isoform
Unigene89595_All	Unigene46169_All	aly/REF export factor
Unigene89595_All	Unigene47476_All	endoglucanase
Unigene89595_All	Unigene48714_All	placenta-specific gene
Unigene89595_All	Unigene48897_All	DENN domain-containing protein
Unigene89595_All	Unigene49495_All	GTP-binding protein
Unigene89595_All	Unigene5198_All	predicted protein
Unigene89595_All	Unigene54753_All	Transposon Ty3-I Gag-Pol polyprotein
Unigene89595_All	Unigene55715_All	RNA-directed DNA polymerase
Unigene89595_All	Unigene58773_All	uncharacterized protein
Unigene89595_All	Unigene58846_All	LDLR chaperone MESD
Unigene89595_All	Unigene60583_All	translation initiation factor
Unigene89595_All	Unigene61677_All	predicted protein
Unigene89595_All	Unigene62595_All	S-adenosylmethionine synthase isoform
Unigene89595_All	Unigene62705_All	uncharacterized protein
Unigene89595_All	Unigene6461_All	transmembrane emp24 domain-containing protein
Unigene89595_All	Unigene65925_All	protein phosphatase 1G
Unigene89595_All	Unigene6864_All	predicted protein
Unigene89595_All	Unigene73569_All	E3 ubiquitin-protein ligase MIB2-like isoform
Unigene89595_All	Unigene7884_All	acyl-CoA-binding domain-containing protein
Unigene89595_All	Unigene80640_All	hypothetical protein
Unigene89595_All	Unigene85463_All	dnaJ homolog subfamily C member
Unigene89595_All	Unigene92314_All	tubulin polyglutamylase TTLL5-like isoform
Unigene9030_All	CL10956.Contig11_All	truncated actin-4
Unigene9030_All	CL10956.Contig6_All	muscle-specific actin-like
Unigene9030_All	Unigene15826_All	hypothetical protein
Unigene9030_All	Unigene57912_All	predicted protein
